# Isotopic constraints on the origin of reactive chlorine in the troposphere

**DOI:** 10.1126/sciadv.aeb5397

**Published:** 2026-01-16

**Authors:** Zheng Zong, Men Xia, Chunshui Lin, Xiaorui Chen, Likun Xue, Qinyi Li, Yifan Jiang, Chongguo Tian, Xuehua Fan, Qi Yuan, Xinfeng Wang, Yujiao Zhu, Jisheng Zhang, Shuncheng Lee, Yujing Mu, Jun Li, Xiao Fu, Chuanhua Ren, Xin Huang, Chao Yan, Wei Nie, Alba Badia, Gan Zhang, Aijun Ding, Ru-Jin Huang, Markku Kulmala, Alfonso Saiz-Lopez, Tao Wang, Wenxing Wang

**Affiliations:** ^1^ Environment Research Institute, Shandong University, Qingdao, Shandong, China.; ^2^ Department of Civil and Environmental Engineering, The Hong Kong Polytechnic University, Hong Kong, China.; ^3^ Institute for Atmospheric and Earth System Research/Physics, Faculty of Science, University of Helsinki, Helsinki, Finland.; ^4^ Aerosol and Haze Laboratory, Beijing Advanced Innovation Center for Soft Matter Science and Engineering, Beijing University of Chemical Technology, Beijing, China.; ^5^ State Key Laboratory of Loess Science, Institute of Earth Environment, Chinese Academy of Sciences, Xi’an, China.; ^6^ School of Atmospheric Sciences, Sun Yat-Sen University, Zhuhai, Guangdong, China.; ^7^ Southern Marine Science and Engineering Guangdong Laboratory (Zhuhai), Zhuhai, Guangdong, China.; ^8^ CAS Key Laboratory of Coastal Environmental Processes and Ecological Remediation, Yantai Institute of Coastal Zone Research, Chinese Academy of Sciences, Yantai, Shandong, China.; ^9^ Shandong Key Laboratory of Coastal Environmental Processes, YICCAS, Yantai, Shandong, China.; ^10^ Key Laboratory of Middle Atmosphere and Global Environment Observation, Institute of Atmospheric Physics, Chinese Academy of Sciences, Beijing, China.; ^11^ Research Center for Eco-Environmental Sciences, Chinese Academy of Sciences, Beijing, China.; ^12^ State Key Laboratory of Organic Geochemistry and Guangdong Key Laboratory of Environmental Protection and Resources Utilization, Guangzhou Institute of Geochemistry, Chinese Academy of Sciences, Guangzhou, Guangdong, China.; ^13^ Institute of Environment and Ecology, Tsinghua Shenzhen International Graduate School, Tsinghua University, Shenzhen, Guangdong, China.; ^14^ Joint International Research Laboratory of Atmospheric and Earth System Research, School of Atmospheric Sciences, Nanjing University, Nanjing, Jiangsu, China.; ^15^ Earth Sciences Department, Barcelona Supercomputing Center, Barcelona 08034, Spain.; ^16^ Institute of Global Environmental Change, Xi’an Jiaotong University, Xi’an, China.; ^17^ Department of Atmospheric Chemistry and Climate, Institute of Physical Chemistry Blas Cabrera, CSIC, Madrid, Spain.

## Abstract

Oceanic and anthropogenic processes, such as sea-salt emissions and combustion activities, release substantial amount of reactive chlorine into the troposphere, affecting air quality, ozone depletion, and climate change. However, distinguishing between these sources for reactive chlorine remains challenging. Here, we establish isotopic constraints on the origin of tropospheric reactive chlorine using chemical ionization mass spectrometry to analyze nitryl chloride (ClNO_2_). Field observations from four regions in China reveal a much broader isotopic value (δ^37^Cl) range for ClNO_2_ (−21 to +39‰) than previously documented for Earth’s chlorine reservoirs. Notably, significant δ^37^Cl differences for ClNO_2_ from sea-salt emissions (−9 ± 4‰) and anthropogenic combustion sources (+20 ± 7‰) were identified. These distinct isotopic signatures, combined with field data, highlight the important role of oceanic chlorine in air pollution, with its chemical cycling affecting not only coastal regions but also extending into inland areas. This research advances our understanding of chlorine’s behavior and cycling in the troposphere.

## Teaser

A large difference in δ^37^Cl between oceanic and anthropogenic origins provides isotopic constraints on tropospheric chlorine.

## INTRODUCTION

Reactive chlorine is an important atmospheric photolabile species that releases highly oxidative chlorine radicals, influencing the Earth’s atmospheric oxidizing power, the fate of various air pollutants and climate-forcing agents, such as ozone (O_3_) and methane (*1*, *2*). Understanding the origin of reactive chlorine is therefore essential for accurately assessing its impact on air quality, ozone depletion, and climate change (*3*). In the troposphere, reactive chlorine primarily originates from sea-salt emissions and anthropogenic combustion activities, including biomass burning, waste incineration, and coal combustion (fig. S1) (*4*, *5*). These oceanic and anthropogenic sources release chlorine mainly as particulate chloride (Cl^−^). Once airborne, these Cl^−^-containing particles undergo transport, deposition, and reactions with nitric acid (HNO_3_) or sulfuric acid (H_2_SO_4_) to produce hydrogen chloride (HCl), and with dinitrogen pentoxide (N_2_O_5_) to produce nitryl chloride (ClNO_2_) or with HO*_x_* radicals to liberate chlorine to other forms of reactive chlorine (*2*, *6*). Interactions between anthropogenic pollution and chloride from these diverse sources frequently lead to elevated concentrations of reactive chlorine species, such as ClNO_2_ and molecular chlorine (Cl_2_), particularly in global coastal regions (*1*, *7*). However, thus far, our understanding of the origin of elevated reactive chlorine remains uncertain due to the absence of precise tracing methods to distinguish between different chlorine sources; for example, chemical transport models, as the mainstream tools, require detailed emission data that are either unavailable or subject to large uncertainties for reactive chlorine species (*8*–*10*).

The stable chlorine isotope ratio (δ^37^Cl), defined as δ^37^Cl = (^37^Cl/^35^Cl)_sample_ / (^37^Cl/^35^Cl)_standard_ − 1, using standard mean ocean chloride (SMOC) as the standard, is a valuable tool for tracing the origin of chlorine on Earth (*11*, *12*). To date, δ^37^Cl values have been extensively reported in various Earth’s reservoirs, such as freshwater, mantle, pore fluid, and oceanic crust (*13*, *14*). Within the troposphere, the δ^37^Cl in the global ocean shows a uniform distribution, nearing 0‰, while marine aerosols exhibit δ^37^Cl values ranging from +0.5 to +2.5‰, and the marine-derived δ^37^Cl-HCl shows depleted values at approximately −1.8‰ (fig. S2) (*15*–*17*). However, δ^37^Cl information for ClNO_2_, anthropogenic Cl^−^ and HCl is still lacking. Detecting δ^37^Cl-ClNO_2_ is highly challenging owing to its photolytic instability, water insolubility, and trace amount; thus, it is undetectable by traditional isotopic methods such as thermal ionization mass spectrometry (TIMS) of the Cs_2_Cl^+^ molecular ion (*18*). The lack of isotopic data for these species limits our understanding of the origin and evolution of tropospheric chlorine and impedes the development of a closed-loop model for the chlorine geochemical cycling, thereby hindering our comprehension of global chlorine dynamics and its impacts (*19*).

In this study, we developed a chlorine isotope method that combines measurements using iodide-adduct time-of-flight chemical ionization mass spectrometer (I^−^-HR-ToF-CIMS) with the sequential Mann-Kendall (SM-K) test to determine the δ^37^Cl-ClNO_2_ in the troposphere and evaluate the anthropogenic δ^37^Cl-Cl^−^ and δ^37^Cl-HCl characteristics (*20*). Building on these isotopic fingerprints, we quantitatively analyzed the interactions of oceanic and anthropogenic chlorine in four representative regions across China. Our results highlight a much broader spectrum of δ^37^Cl values within the Earth system than previously documented, contrasting δ^37^Cl disparities between the oceanic and anthropogenic sources, and the extensive transport of ocean-derived chlorine to interior continent via atmospheric chlorine cycling. By establishing isotopic constraints, our research enhances the understanding of chlorine’s behavior and cycling in the troposphere.

## RESULTS

### Wide range of δ^37^Cl-ClNO_2_ and its diversity in the troposphere

**Fig. 1.**
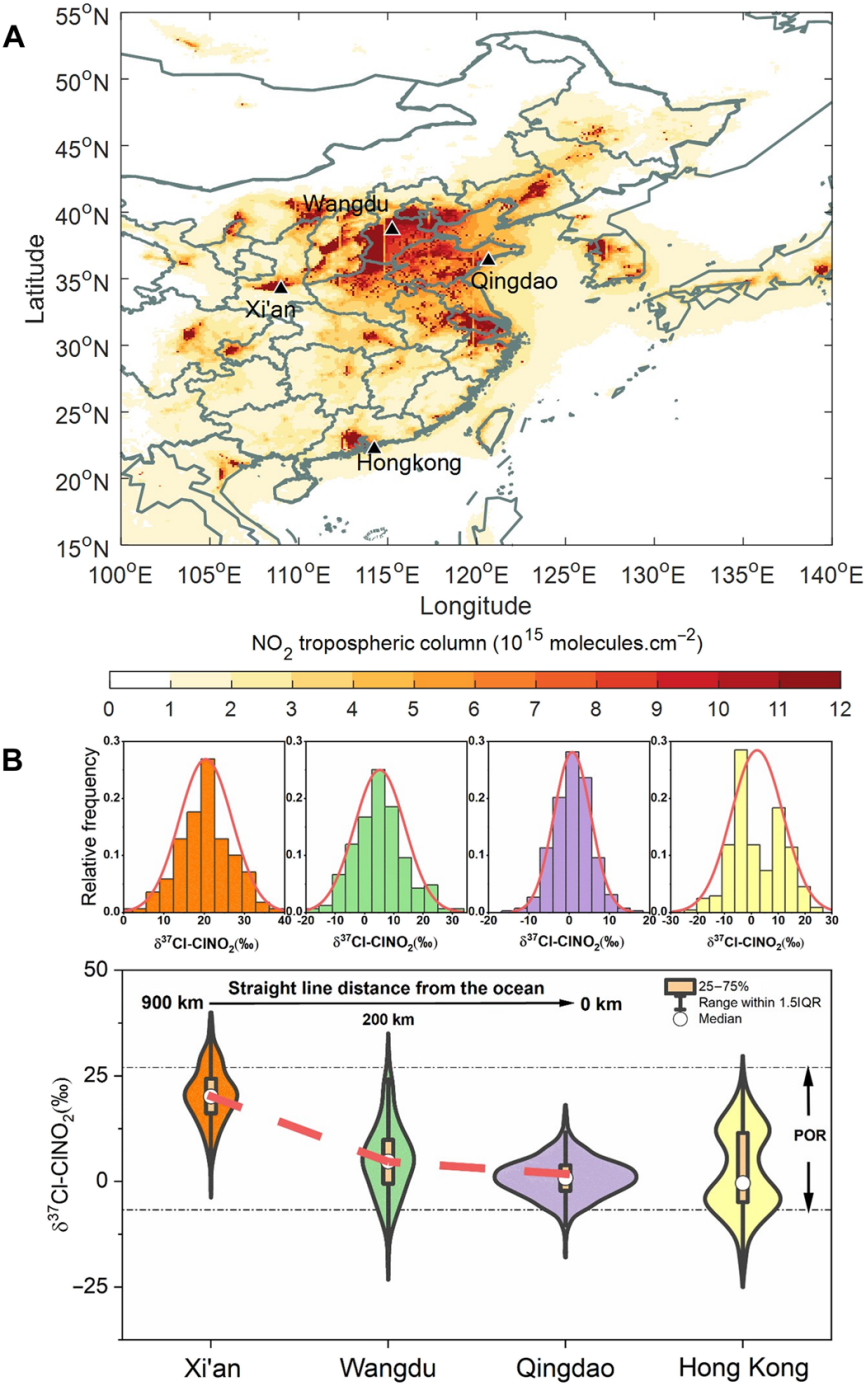
Spatial distribution of δ^37^Cl-ClNO_2_ in four representative regions across China. (**A**) Geographical locations of the four study sites. The color code represents the average NO_2_ column derived from the TROPOMI satellite retrieval during the observations, which indicates the level of nitrogen pollution affecting atmospheric chlorine activity. (**B**) δ^37^Cl-ClNO_2_ characteristics of the four sites, exhibiting normal distributions, with values decreasing as the distance from the ocean decreases, as deduced from Xi’an, Qingdao, and Wangdu; these areas are located in regions at similar latitudes and with comparable anthropogenic chlorine emissions. The distribution of each group in the violin plot is shown by a kernel density plot, with the width indicating the data density at that value. The term “POR” refers to the previously observed range of δ^37^Cl signatures across the Earth system, as shown in fig. S4.

To explore the δ^37^Cl characteristics of reactive chlorine in the troposphere, we conducted comprehensive field observations specifically targeting ClNO_2_, in four representative regions of China: Xi’an (West China; inland), Wangdu (North China; inland), Qingdao (East China; coastal), and Hong Kong (South China; coastal) (Fig. 1A and Materials and Methods). The mixing ratios of ClNO_2_ during the observation periods were 49 parts per trillion by volume (pptv; 10 to 170), 37 pptv (9 to 145), 212 pptv (63 to 562), and 165 pptv (15 to 408) for Xi’an, Wangdu, Qingdao, and Hong Kong, respectively, based on the 5-min median and the 25th to 75th percentiles (fig. S3). These ClNO_2_ levels are comparable to or greater than those measured in North America and Europe, showing large chlorine emission and active chlorine chemistry across China (*3*, *21*). In addition, the concentrations of ClNO_2_ in the coastal areas (Qingdao and Hong Kong) were significantly higher than those in inland locations (Wangdu and Xi’an; *P* < 0.01, Student’s *t* test), and this is primarily attributed to the additional input of oceanic Cl^−^ in the coastal areas (*1*).

Here, we developed a method that employs an iterative SM-K test to analyze the ^37^Cl and ^35^Cl signals obtained from I^−^-HR-ToF-CIMS, enabling the extraction of the δ^37^Cl-ClNO_2_ values (Materials and Methods). During the four observations, the determined δ^37^Cl-ClNO_2_ values exhibited a wide range, varying from −21 to +39‰. This δ^37^Cl-ClNO_2_ range largely exceeds the previously known δ^37^Cl signatures across the entire Earth system, such as those in the troposphere (−3.5 to +2.5‰, encompassing δ^37^Cl-Cl^−^ of oceanic aerosols and marine δ^37^Cl-HCl), groundwater (−1.5 to +1.6‰) and volcanic fumaroles (−2.8 to +14‰) (fig. S4) (*16*–*18*). Before this study, the highest observed δ^37^Cl record of the Earth system was from CF_2_Cl_2_ in the stratosphere, reaching +27‰, which was primarily attributed to isotopic fractionation during the photolytic and chemical decomposition of CF_2_Cl_2_ (*22*). Similarly, we confirm that the wide range of δ^37^Cl-ClNO_2_ values partly results from the isotopic fractionation during ClNO_2_ formation, and this aspect is addressed in the following sections. These isotopic observations clearly represent a substantial expansion of the chlorine isotope range within the Earth system, indicating pronounced fractionation of reactive chlorine during solid-gas phase transitions in the troposphere. This finding provides isotopic evidence and theoretical constraints for evaluating the atmospheric chemical processes and circulation mechanisms governing reactive chlorine.

**Fig. 2.**
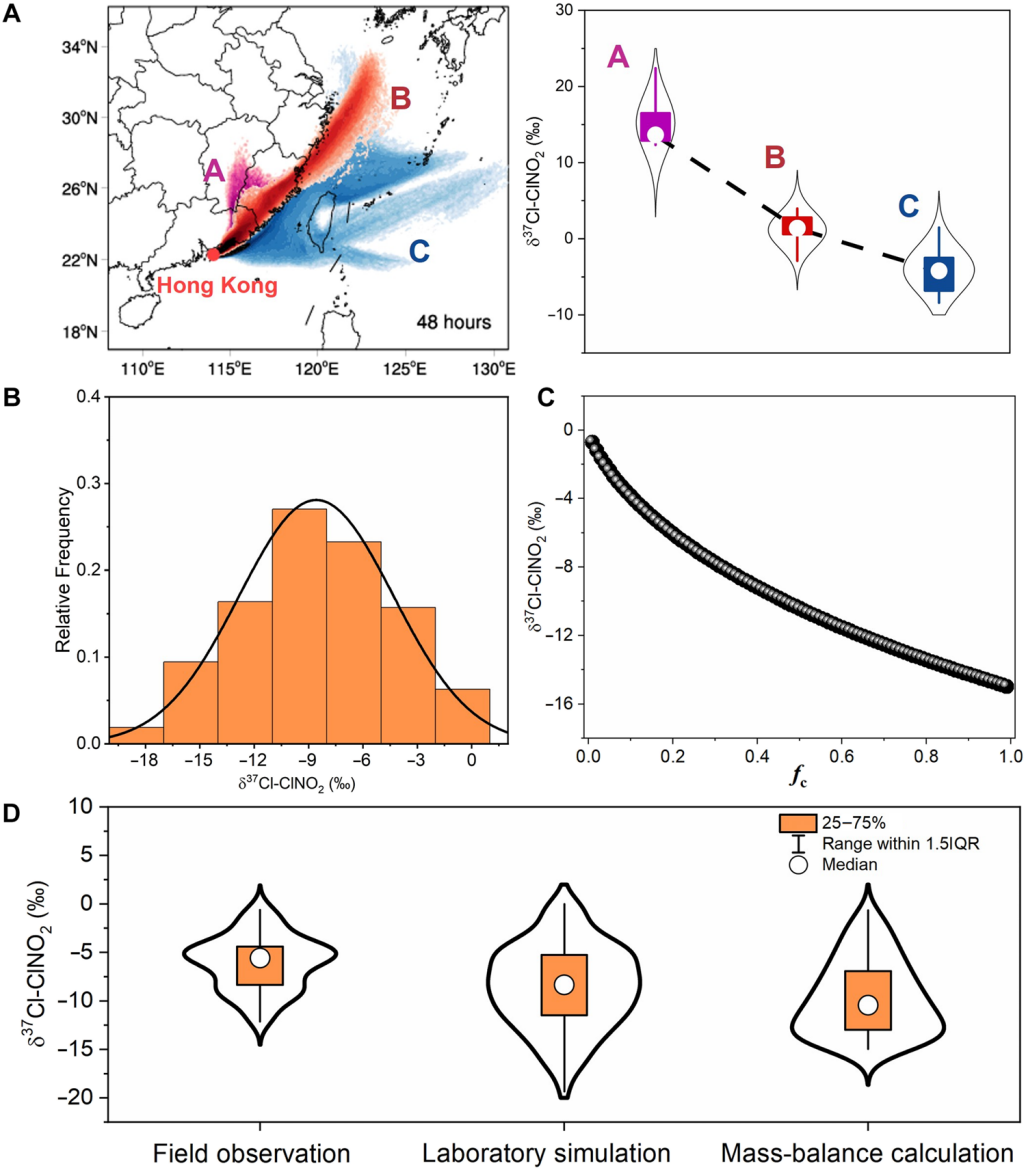
Characteristics of the oceanic δ^37^Cl-ClNO_2_ obtained through three independent methods, i.e., field observations, laboratory experiments, and mass-balance calculations. (**A**) Field observations of the hourly δ^37^Cl-ClNO_2_ characteristics combined with 48-hour LPDM backward trajectories in Hong Kong; these results indicate a notable variation in δ^37^Cl-ClNO_2_ in different air masses. (**B**) Statistical analysis of the oceanic δ^37^Cl-ClNO_2_ values derived from fresh sea-salt particles reacting with N_2_O_5_ in laboratory experiment, with a normal distribution. (**C**) Nonlinear relationship between oceanic δ^37^Cl-ClNO_2_ and *f*_c_ obtained through mass-balance calculations. *f*_c_ is the fraction of unreacted Cl^−^ remaining during its reaction with N_2_O_5_ to form ClNO_2_. (**D**) Violin plots of oceanic δ^37^Cl-ClNO_2_ datasets derived from field observations, laboratory simulations, and mass-balance calculations.

Our study focuses on the δ^37^Cl-ClNO_2_ of oceanic and anthropogenic origins. As depicted in Fig. 1B, a noticeable geographical trend in δ^37^Cl-ClNO_2_ is observed, with a clear decrease from the inland regions toward the coastal areas. Specifically, Xi’an (average ± SD: +20 ± 7‰), Wangdu (+5 ± 9‰), and Qingdao (+1 ± 5‰) stand out in this trend. These three sites are located in regions at similar latitudes and with comparable levels of anthropogenic chlorine emissions (fig. S5) (*4*). In addition, the violin plot for Hong Kong exhibits bimodal peaks in δ^37^Cl-ClNO_2_ (Fig. 1B), primarily resulting from the interaction between oceanic and continental air masses (*23*). As depicted in Fig. 2A, continent-derived air masses are predominantly influenced by anthropogenic chlorine emissions, which display significantly higher δ^37^Cl-ClNO_2_ values than oceanic sources such as sea-salt emissions. Consequently, the bimodal δ^37^Cl-ClNO_2_ distribution in Hong Kong reflects shifts in atmospheric reactive chlorine sources driven by variations in air mass transport, further highlighting the distinct isotopic signatures of oceanic and anthropogenic chlorine.

### Differences in δ^37^Cl between the oceanic and anthropogenic chlorine compounds

We then used three independent methodologies to determine the authentic oceanic δ^37^Cl-ClNO_2_ signatures; these methods included field observations, laboratory experiments, and mass-balance calculations (Materials and Methods). Building on field observations at the coastal site of Hong Kong, we filtered out the measurement data representative of oceanic air according to the backward trajectories computed by the Lagrangian particle dispersion model (LPDM; fig. S6) (*23*). This yielded an average δ^37^Cl-ClNO_2_ value (±SD) of −6 ± 3‰, with a range extending from −12 to −1‰ (Fig. 2A). We also generated pure oceanic ClNO_2_ by reacting N_2_O_5_ with fresh marine aerosols derived from real seawater in a smog chamber and analyzed the δ^37^Cl-ClNO_2_ signals (*24*). This direct determination revealed that the oceanic δ^37^Cl-ClNO_2_ values varied from −19 to 0‰, with an average of −9 ± 4‰ (Fig. 2B). We further used the available δ^37^Cl-Cl^−^ data of oceanic aerosols to conduct mass-balance calculations of the oceanic δ^37^Cl-ClNO_2_; these data were previously obtained using the traditional TIMS technique (*16*). The estimated range extended from −15 to −1‰, with the fraction of chlorine remaining in the condensed phase after reactions between fresh sea salt and N_2_O_5_ (*f*_c_) varying from 0.01 to 0.99 (Fig. 2C) (*25*, *26*). No significant difference was observed among the three datasets derived from the field observations, laboratory experiments, and mass-balance calculations (*P* > 0.05, Student’s *t* test; Fig. 2D), thereby validating the robustness of our oceanic δ^37^Cl-ClNO_2_ determination. These results clearly indicate that oceanic ClNO_2_ exhibits an overall negative δ^37^Cl value due to isotopic fractionation. On the basis of the laboratory experiments, which are unaffected by atmospheric transport and other external processes, the oceanic δ^37^Cl-ClNO_2_ value was estimated to be −9 ± 4‰. Notably, seawater δ^37^Cl values exhibit homogeneity globally (*24*); therefore, our confirmed oceanic δ^37^Cl-ClNO_2_ values could be considered globally representative.

The δ^37^Cl-ClNO_2_ data collected in Xi’an (+20 ± 7‰) are representative of the anthropogenic Cl^−^ sources, which is supported by the predominant origin of air masses from inland regions during the observations (fig. S7) (*27*). A significant contrast is observed in the δ^37^Cl signals between the anthropogenic (positive) and oceanic (negative) ClNO_2_, with discrepancies of up to 29‰. This important finding provides isotopic constraints to better understand the origins of ClNO_2_ (*28*). Specifically, higher positive δ^37^Cl-ClNO_2_ values indicate substantial chlorine input from anthropogenic sources, whereas lower negative values reveal a dominant oceanic origin. The disparity exhibited by δ^37^Cl highlights its comparable potential to those of other isotopic tracers (e.g., δ^13^C, δ^15^N, and δ^34^S) in addressing the sources and formation mechanisms of air pollution (*29*). The substantial difference in isotopic signatures between oceanic and anthropogenic sources also significantly contributes to the broad range of δ^37^Cl values observed in atmospheric ClNO_2_.

**Fig. 3.**
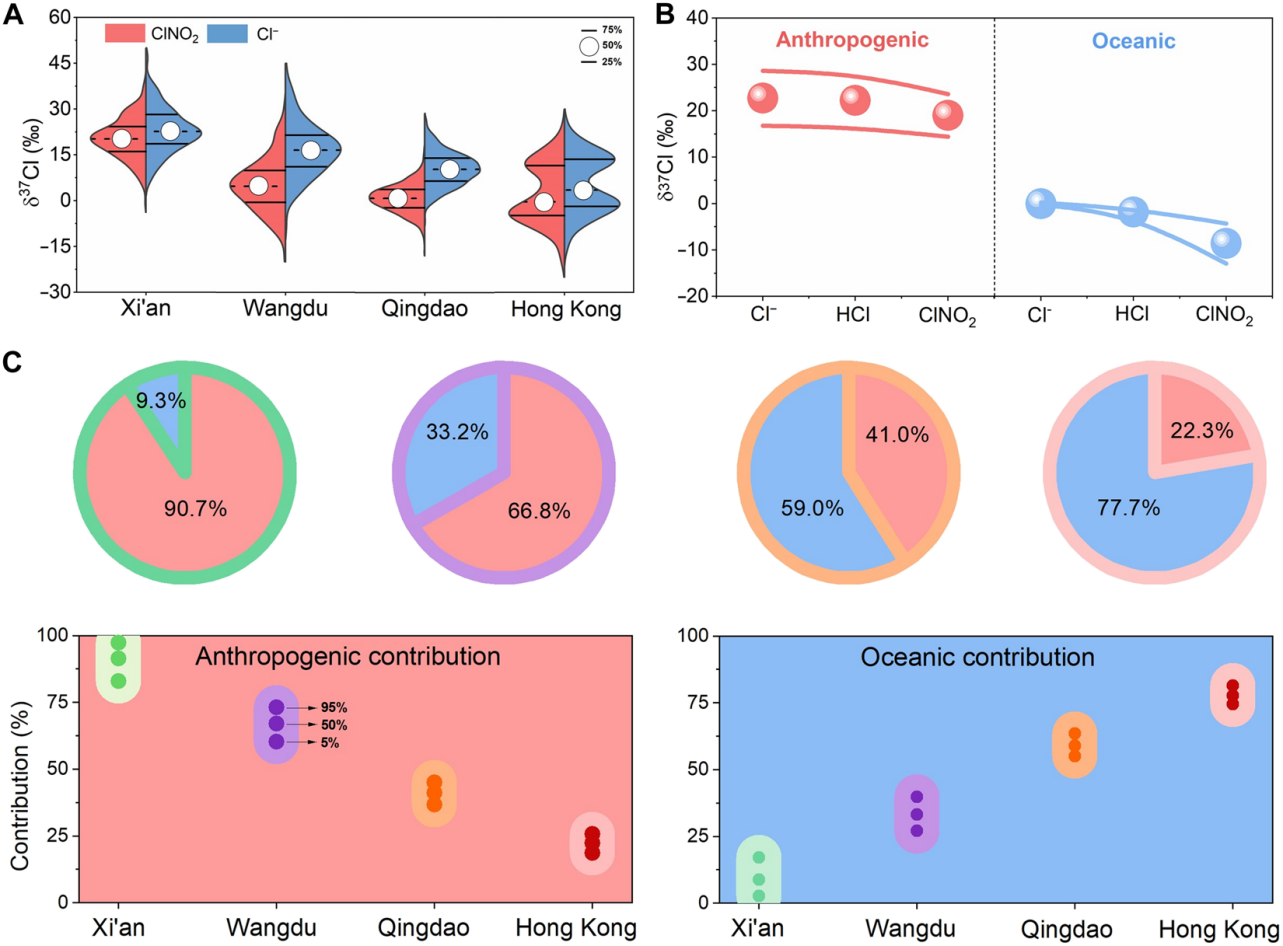
Isotopic characteristics of the key atmospheric chlorine species (Cl^−^, HCl and ClNO_2_) and their source contributions at the four sites. (**A**) Comparative isotopic signatures of ClNO_2_ and its Cl^−^ precursor, where their differences are caused by isotopic fractionation. (**B**) δ^37^Cl values of the major chlorine species, including Cl^−^, HCl, and ClNO_2_, which show significant differences between the oceanic and anthropogenic origins. In the visual representation, the dot indicates the average, and the line represents the SD. (**C**) Contributions of the oceanic and anthropogenic sources to atmospheric chlorine species in Xi’an, Wangdu, Qingdao, and Hong Kong. The three points represent the 5th, 50th, and 95th percentiles of the contributions.

To confirm the ubiquity of the isotopic disparities among atmospheric chlorine species (i.e., Cl^−^ and HCl) of oceanic and anthropogenic origins, we conducted an analysis of isotope fractionation during the conversion reactions of Cl^−^ to HCl and ClNO_2_ (Materials and Methods) (*16*). This fractionation showed a significant nonlinear correlation with the *f*_c_ (*P* < 0.01, Student’s *t* test) (*30*, *31*). On the basis of this insight, we quantified the magnitude of isotope fractionation in the ClNO_2_ formation of the study regions. Wangdu exhibited the most significant fractionation (−12 ± 1‰), followed by Qingdao (−10 ± 2‰), Hong Kong (−3 ± 2‰), and Xi’an (−3 ± 3‰). The variation in isotope fractionation across these regions was primarily attributed to the substantial differences in *f*_c_, which is affected by factors such as concentrations of Cl^−^ (fig. S8) and N_2_O_5_ (*16*, *32*). These results highlight the significant regional variability in chlorine fractionation during ClNO_2_ formation, which accounts for the observed broad range of δ^37^Cl-ClNO_2_ values. In addition, we found that isotopic fractionation between Cl^−^ and HCl in the atmosphere is significantly lower than that between Cl^−^ and ClNO_2_, with a fractionation range of approximately 0 to 2‰. This finding aligns with previous theoretical calculations, further confirming the accuracy of our results (*33*). With these fractionation characteristics and the measured δ^37^Cl-ClNO_2_, we further calculated the δ^37^Cl-Cl^−^ and δ^37^Cl-HCl values for the four study regions (i.e., δ^37^Cl-Cl^−^; Fig. 3A). Notably, similar to the trend observed for δ^37^Cl-ClNO_2_, the δ^37^Cl-Cl^−^ and δ^37^Cl-HCl values in these regions also exhibit a decreasing trend from inland to the coastal regions.

The δ^37^Cl values for anthropogenic Cl^−^ and HCl (~+23‰; Fig. 3B) were estimated on the basis of measurements of δ^37^Cl-ClNO_2_ and isotope fractionation factors in Xi’an. These elevated δ^37^Cl values closely match those observed for waste incineration (δ^37^Cl-Cl^−^: +17 ± 9‰), a major anthropogenic source identified using traditional TIMS analysis (Materials and Methods) (*4*, *34*). We suggest that during high-temperature combustion process (e.g., waste incineration, coal combustion, and biomass burning), the lighter ^35^Cl preferentially transitions into the gas phase, while the heavier ^37^Cl tends to remain in particle-phase chloride (*31*). This fractionation leads to the enrichment of δ^37^Cl in Cl^−^, the direct precursor of ClNO_2_. As illustrated in Fig. 3B, large disparities existed across all three chlorine species, with δ^37^Cl values from anthropogenic sources significantly higher than those from oceanic sources. Furthermore, significant differences were also observed among the δ^37^Cl values of HCl, ClNO_2_, and Cl^−^ from the same source, particularly for oceanic chlorine (e.g., δ^37^Cl-Cl^−^ > δ^37^Cl-ClNO_2_). These results established a basis for the potential application of isotope-based techniques to trace the origins and evolution of atmospheric chlorine. For instance, both Cl^−^ and ClNO_2_ serve as precursors for Cl_2_, resulting in distinct isotopic signatures in the produced Cl_2_ (*35*). Future determination of δ^37^Cl-Cl_2_ will enable quantification of these Cl_2_ formation pathways and broad application of atmospheric chlorine isotopes.

### Extensive ocean-continent interactions of atmospheric chlorine

On the basis of the difference in δ^37^Cl between the oceanic and anthropogenic origins and the Bayesian model (Materials and Methods) (*19*), we quantified the contributions of the oceanic and anthropogenic sources to the ClNO_2_ during periods when concentrations were sufficiently high for isotopic analysis (*22*). Hong Kong exhibited the highest contribution from oceanic sources (77.7 ± 19.0%), followed by Qingdao (59.0 ± 2.5%) and Wangdu (33.2 ± 3.9%), while Xi’an showed the lowest but still appreciable oceanic contribution (9.3 ± 4.4%; Fig. 3C). This trend was consistent with the established understanding of the atmospheric influence of oceanic sources on the continent; the impact progressively diminished with increasing distance inland (*21*). Nevertheless, the extensive reach of oceanic influence at Wangdu was noteworthy; its location was approximately 200 km inland from the nearest ocean, and the observations were conducted during the cold season (February-March) when the inland northwesterly winds prevailed. Oceanic Cl^−^ originates from sea-salt particles, which are generated through physical processes and are primarily coarse (*36*). Therefore, the influence of oceanic chlorine was assumed to be predominantly confined to coastal regions. However, our isotopic observations revealed a more substantial contribution of oceanic chlorine to the inland regions, and these results were further supported by the LPDM analysis (text S1 and figs. S7 and S9) (*37*).

**Fig. 4.**
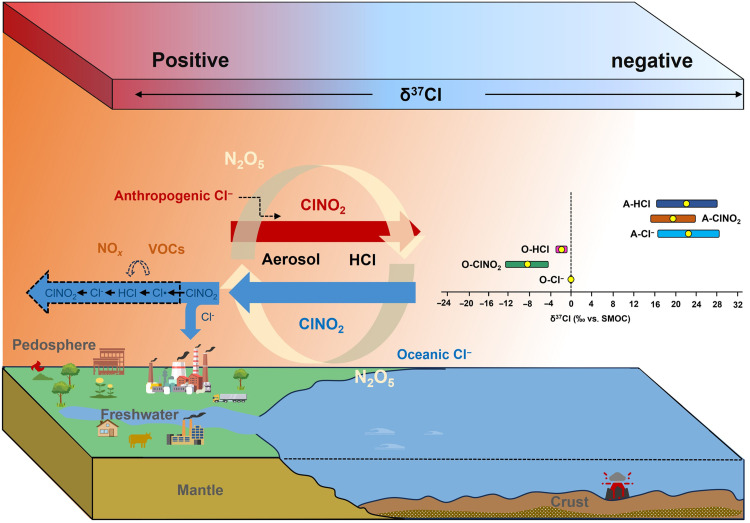
Isotopic constraints on the cycling of chlorine in the troposphere. The panel illustrating isotopic distribution characteristics shows the range as the average ± SD, with the yellow dots denoting the mean values. The abbreviations “A” and “O” represent anthropogenic and oceanic origins, respectively. The orange background qualitatively indicates the levels of anthropogenic pollution (e.g., NO*_x_* and VOCs), with darker colors signifying higher concentrations. In continental regions with elevated anthropogenic pollution, oceanic Cl^−^ can prolong its lifetime and expand its influence through atmospheric chemical age (e.g., coarse-mode Cl^−^ → ClNO_2_ → Cl radical → HCl → fine-mode Cl^−^ → ClNO_2_).

We speculate that the residence lifetime and transport distance of oceanic Cl^−^ can increase through atmospheric chemical cycling (e.g., coarse-mode Cl^−^ → ClNO_2_ → Cl radical → HCl → fine-mode Cl^−^ → ClNO_2_; table S1) (*2*), particularly in regions with high levels of anthropogenic pollutants, such as NO*_x_* and volatile organic compounds (VOCs) (Fig. 4). To validate this mechanism, we used a weather research and forecasting model coupled with chemistry (WRF-Chem; Materials and Methods and table S2), which incorporated the latest reactive chlorine chemistry, and a sensitivity analysis with only oceanic sources of chlorine, including both coarse- and fine-mode chloride, was conducted (*38*). The simulation results clearly indicated that oceanic chlorine (Cl*_y_*) could extend far inland (fig. S10), covering distances exceeding 1000 km, which was consistent with the isotopic results in this study (e.g., Xi’an, approximately 1000 km from the ocean, accounted for approximately 9% of the oceanic contribution). In addition, as chlorine penetrated deeper into the continent, gaseous chlorine species became overwhelmingly prominent, providing further support for the above proposed cycling mechanism. This mechanism persisted even during the cold season when inland winds prevailed, resulting in the widespread presence of oceanic chlorine in continental regions (*39*). Recent modeling work also supports this oceanic chlorine transport mechanism, finding ClNO_2_ levels up to 200 pptv in air masses extending 600 km inland from the coast (*40*). The result provides independent evidence that marine-derived chlorine can undergo long-range transport and exert a substantial impact on inland atmospheric chemical processes.

The contribution of anthropogenic chlorine to ClNO_2_ in Qingdao was 41.0 ± 2.5%, which was much greater than that in Hong Kong (22.3 ± 1.9%), despite both locations being near the seaside. This difference highlights the influence of anthropogenic emissions in East China on atmospheric chlorine activation (*41*). Examination of the forward trajectories of the air masses at the two sites revealed that these anthropogenic chlorines infiltrated the oceanic regions, participating in atmospheric chemical cycling until they were deposited onto the oceanic surface (e.g., Qingdao; fig. S11). Furthermore, contributions exceeding 20% showed the significant impacts of anthropogenic chlorine on offshore air quality and even marine air quality, especially in conjunction with elevated concentrations of reactive nitrogen oxides and VOCs within continental air masses (*42*).

## DISCUSSION

In this study, we develop an isotopic method to measure the isotopic signatures of ClNO_2_ and infer those of anthropogenic HCl and Cl^−^. We found a much broader range of δ^37^Cl values within the Earth system than previously documented; these results could improve our comprehension of the tropospheric chlorine cycle and facilitate comparisons with other Earth’s chlorine reservoirs (*14*). Specifically, this broad range is primarily attributed to the notable isotopic fractionation during the conversion of particulate Cl^−^ to gaseous reactive chlorine and the previously unrecognized high δ^37^Cl values of continental anthropogenic emissions. This finding enhances our understanding of the chlorine isotopic fractionation during its migration and transformation in the troposphere. It also strengthens our ability to trace anthropogenic chlorine as it enters different environmental subsystems, such as the cryosphere and freshwater, through its distinct isotopic signatures (*13*).

The contrasting disparities in δ^37^Cl between the oceanic and anthropogenic sources, as revealed in our study, enable the application of chlorine isotopes in distinguishing chlorine sources in regions where oceanic and continental influences converge (*1*). On the basis of this disparity, we determined the capacity of ocean-derived chlorine to prolong its atmospheric lifespan and transport distance to inland regions. This finding highlights the influence of oceanic conditions on continental air quality, particularly concerning the role of reactive chlorine from oceanic Cl^−^ in secondary air pollution formation, including the enhancement of O_3_, nitrate and organic aerosols (*43*). Similarly, chlorine isotopes emitted by various anthropogenic sources, such as biomass burning and coal combustion, will likely exhibit differences. These results indicate the potential utility of chlorine isotopes in tracing the elevated concentrations of reactive chlorine observed in continental regions; in addition, in conjunction with other isotopes, this can aid in the resolution of complex air pollution involving multiple elements (e.g., C, N, Cl, S, and O) in heavily polluted areas (*29*).

In summary, we now have an isotopic methodology to retroactively trace the oceanic and anthropogenic sources of reactive chlorine within the troposphere. However, this method currently requires sufficient ambient ClNO_2_ concentrations, limiting its application to environments where precursors like NO*_x_* and O_3_ are abundant enough to generate adequate N_2_O_5_ (*44*). Future advancements in analytical techniques are expected to overcome this limitation by enabling isotopic measurements at lower concentrations and for a broader range of reactive chlorine species. This optimism is supported by the fact that the detection limit of the iodide-adduct long time-of-flight chemical ionization mass spectrometer (I^−^-HR-LToF-CIMS) is 1.5 times lower than that of the I^−^-HR-ToF-CIMS (fig. S12). Furthermore, our findings underscore the pivotal role of oceanic chlorine in shaping air pollution and tropospheric chemistry, not only in coastal areas but also in inland regions where strong anthropogenic emissions prevail. This connection has likely been previously underestimated and should be carefully considered when assessing air quality, atmospheric processes, and their broader environmental and health implications.

## MATERIALS AND METHODS

### Field measurements

To investigate the chlorine isotopic characteristics of ClNO_2_ in the troposphere, we conducted comprehensive field campaigns during cold seasons at four distinct sites across China: Wangdu in North China, Qingdao in East China, Hong Kong in South China, and Xi’an in West China (Fig. 1A and text S2). These locations were selected considering the influence of the atmospheric chlorine activity caused by the high concentrations of reactive nitrogen oxides (e.g., NO_2_ and N_2_O_5_), along with factors such as the distance from oceans to continents and variations in chlorine emissions (*45*).

Specifically, the field campaign in Wangdu occurred from 11 February to 6 March 2023, at the Station of Rural Environment of Research Center for Eco-Environmental Sciences of Chinese Academy of Sciences, which is located in Dongbaituo village (38.66°N, 115.25°E). This village is surrounded by agricultural fields planted with winter wheat and is approximately 200 km from the ocean and 160 km from Beijing, the capital of China. The Qingdao field campaign was carried out from 1 December to 31 December 2022, on the roof of a four-story building near the Qingdao campus of Shandong University (36.35°N, 120.67°E), 40 km northeast of downtown Qingdao and 1 km north of the coastline. The field campaign in Hong Kong occurred from 6 October to 24 November 2020, at the Cape D’Aguilar supersite (22.21°N, 114.25°E), which is situated at the southeast tip of Hong Kong Island. Cape D’Aguilar features a rural coastal setting on a hill surrounded by vegetation and sparse country roads and is approximately 10 km from downtown Hong Kong. The field campaign in Xi’an was conducted from 10 March to 9 April 2022, at the Qin Ling supersite (34.07°N, 108.35°E), approximately 80 km southwest of downtown Xi’an. This site serves as a regional background site. These four stations were extensively used to investigate the regional characteristics of air pollution and atmospheric chemistry processes in China, and detailed descriptions can be found elsewhere (*35*, *42*, *46*).

ClNO_2_ was measured by an I^−^-HR-ToF-CIMS (Aerodyne Research Inc., USA) in Wangdu, Qingdao, and Hong Kong, while the observation in Xi’an was based on an upgraded version of the instrument (I^−^-HR-LToF-CIMS; Aerodyne Research Inc., USA) (*47*). In brief, I^−^ and its water cluster (IH_2_O^−^) produced by ionization of CH_3_I gas were used as the reagent ions. ClNO_2_ combined with I^−^ or IH_2_O^−^ to produce IClNO_2_^−^, which was detected at 208 atomic mass unit [amu; I^35^ClNO_2_^−^, exact mass: mass/charge ratio (*m*/*z*) 207.8668] and 210 amu (I^37^ClNO_2_^−^, exact mass: *m*/*z* 209.8639), respectively. The signals of I^35^ClNO_2_^−^ and I^37^ClNO_2_^−^ were independently obtained by Tofware version 3.0 (the data processing software) using high-resolution peak fitting at the respective nominal masses without any constraint of the isotopic signals for I^37^ClNO_2_^−^ (fig. S13). For details regarding the instrument configuration and calibrations, please refer to the text S2. In addition, the Cl^−^ concentrations were determined in parallel using an online ion chromatography in Wangdu, Qingdao, and Hong Kong and a LToF aerosol mass spectrometer in Xi’an (Aerodyne Research Inc., USA) (*48*).

### Laboratory experiments

To directly quantify the δ^37^Cl of oceanic ClNO_2_, we conducted simulations of ClNO_2_ formation from reactions of N_2_O_5_ and sea spray in a Teflon smog chamber (volume, 5.4 m^3^) at the Hong Kong Polytechnic University, and measured the δ^37^Cl-ClNO_2_ values via I^−^-HR-ToF-CIMS (*49*). The specific experimental design is shown in fig. S14 and is described as follows. Before the experiment, the smog chamber underwent a 48-hour flush with zero air generated by a zero-air generator (Model 111, Thermo Fisher Scientific Inc., USA) to reduce the background levels of ClNO_2_ and other related species. O_3_ produced by an ozone generator (Ozone Generator Model 2000, Jelight Company Inc., USA) was introduced into the smog chamber at a controlled mixing ratio of 250 ppbv. After achieving uniform mixing (~20 min), NO_2_ (10 ppmv in a gas cylinder, Scientific Gas Inc., Australia) was injected into the chamber, reaching a mixing ratio of 150 ppbv by controlling the flow rate and time. To ensure the rapid generation of a substantial amount of N_2_O_5_, a reaction time of 10 min was used, as suggested by a previous study (*49*). Subsequently, fresh sea-salt particles generated by an aerosol generator (Aerosol Generator 3079A, TSI Inc., USA) with seawater were introduced into the smog chamber to produce oceanic ClNO_2_ (fig. S15). The seawater used for generating fresh sea-salt particles was collected near the Hong Kong site to better represent the characteristics of its oceanic air masses during field observations (*35*). The seawater was thermally concentrated three times to ensure an ample supply of ClNO_2_ for isotopic analysis.

Throughout the experiment, we maintained the temperature and humidity of the smog chamber at 25°C and 35%, respectively, in the dark. The concentrations of NO_2_ and O_3_ were continuously monitored using an NO*_x_* analyzer equipped with a blue light converter (Model 42i-TL, Thermo Fisher Scientific Inc., USA) and an O_3_ analyzer (Model 49i, Thermo Fisher Scientific Inc., USA), respectively. Real-time measurements of ClNO_2_ and N_2_O_5_ were conducted using the same high-resolution I^−^-HR-ToF-CIMS used in the field observations, with N_2_O_5_ quantified at 235 amu (IN_2_O_5_^−^). The I^−^-HR-ToF-CIMS underwent calibration using the same procedure as that used in the field campaigns (*47*). As depicted in fig. S15, a substantial amount of N_2_O_5_ was generated before the introduction of sea spray, yet the mixing ratio of ClNO_2_ remained very low. This observation indicated that the smog chamber exhibited minimal background levels of ClNO_2_.

### Chlorine isotopic detection of fly ash samples from waste incineration

Fly ash from waste incineration typically contains high concentrations of water-soluble chlorine, making it well-suited for traditional chlorine isotope analysis (*50*). Moreover, atmospheric chlorine emission inventories identify waste incineration as a major anthropogenic chlorine source (*4*). To verify the isotopic composition of anthropogenic Cl^−^ inferred in this study, we collected fly ash samples from waste incineration sites in Xi’an (10 samples) and Shaoxing, Zhejiang Province (three samples) and analyzed them using the traditional TIMS method (*34*). The analysis involved grinding the fly ash samples, dissolving them in ultrapure water, and filtering the solution to extract Cl^−^. Chloride ions were enriched through ion exchange and converted into Cs_2_Cl^+^ molecular ions to reach the concentration required for detection. The Cs_2_Cl^+^ ions were then analyzed using a Triton thermal ionization mass spectrometer, with SMOC as the reference standard. Parallel samples were tested to evaluate measurement uncertainty, which was found to be less than 0.5‰ (*15*, *51*). The δ^37^Cl value of Cl^−^ in the Xi’an samples was +17.7 ± 10.0‰, while the Shaoxing samples measured +15.5 ± 3.4‰. These values are significantly higher than those of oceanic Cl^−^ (~0‰) but closely align with the anthropogenic sources inferred in this study (fig. S16). The slight discrepancies may reflect the composite nature of the inferred value, which integrates emissions from diverse sources (e.g., waste incineration and biomass burning) (*44*).

Waste incineration typically involves the combustion of a complex mixture of materials, such as plastics (e.g., polyvinyl chloride), many of which contain substantial amounts of organochlorine compounds, often exceeding 80% of the total chlorine content (*52*). Although the chlorine isotopic composition of these organochlorine components has not yet been reported, previous studies have shown that δ^37^Cl values for synthetic chlorinated ethylene and their degradation products range from −3.7 to 10.7‰ (fig. S4) (*53*, *54*). On the basis of this range, we selected an approximate median value of 5‰ as the initial parameter of waste incineration considering their similar formation processes to chlorinated ethylene. The dechlorination process during incineration can be broadly divided into two thermal stages. In the first stage (around 200°C), organochlorines undergo dehydrochlorination, producing gaseous HCl (*52*). In the second stage (above 700°C), the remaining organochlorines are almost completely converted to particulate Cl^−^, with a conversion efficiency exceeding 98%. The isotopic fractionation associated with these processes can also be divided into two corresponding phases. During the first stage, the isotopic fractionation follows Rayleigh-type behavior. The residual organochlorine isotope composition can be described by

$$\delta^{37}{\mathrm{Cl}}_{\text{remaining}}=\delta^{37}{\mathrm{Cl}}_{0}+\left(\alpha -1\right)\times \text{ln}\left(f_{c}\right)\times 1000$$ (1)

where δ^37^Cl_0_ is the initial isotopic composition of the organochlorines, α is the isotopic fractionation factor associated with the transformation from organochlorine to HCl, and *f*_c_ is the fraction of chlorine retained in the condensed phase (*16*). On the basis of transition state theory (*55*), the fractionation factor α can be estimated as

$$\alpha =K_{37}/K_{35}=1/\text{exp}\left[\left(\Delta m/m\right)\left(E_{a}/\mathrm{RT}\right)\right]$$ (2)

Here, Δ*m*/*m* is the relative mass difference between ^37^Cl and ^35^Cl, *Ea* is the activation energy (assumed to be ~375 kJ/mol for C─Cl bond cleavage), *R* is the universal gas constant, and *T* is the reaction temperature. At 200°C, α is estimated to be approximately 0.9946. Using a chlorine emission ratio of HCl to Cl^−^ of about 3:1 (as reported in the emission inventory in China) (*4*), the fraction of chlorine retained in the condensed phase (*f*_c_) is estimated at 0.25. Substituting these values into the Rayleigh equation yields a δ^37^Cl value of approximately +12.5‰ for the residual organochlorine. Notably, this estimate is likely conservative. Some studies report HCl formation rates as high as 95%, implying a much lower *f*_c_ (e.g., 0.05) (*52*), which would result in even higher δ^37^Cl values, up to +21.2‰. In the second stage of incineration, virtually all remaining organochlorines are converted to Cl^−^, and the associated isotope fractionation is considered negligible. Therefore, the final δ^37^Cl value of Cl^−^ in fly ash reflects the residual signal established during the initial dechlorination stage. This theoretical average value (~+12.5‰) is consistent with our measured results for δ^37^Cl values of Cl^−^ in fly ash from waste incineration. It is important to acknowledge, however, that after emission into the atmosphere, both Cl^−^ and HCl may undergo isotopic equilibrium fractionation (*31*). This process tends to reduce the δ^37^Cl value of Cl^−^ to some extent, although it generally retains a relatively enriched isotopic signature (text S3). Such enrichment serves as a distinctive isotopic tracer, enabling differentiation between chlorine originating from anthropogenic combustion sources and that from marine sources, which are typically characterized by lower δ^37^Cl values.

### Extraction of reliable δ^37^Cl-ClNO_2_ data based on the SM-K test

In this study, we developed a method that could extract δ^37^Cl-ClNO_2_ from the ^37^Cl and ^35^Cl signals obtained from I^−^-HR-ToF-CIMS based on an iterative SM-K test analysis (*20*). Here, we used the measurement data in Hong Kong as an example to elucidate the steps and procedures involved in extracting reliable δ^37^Cl-ClNO_2_ data. Figure S17 shows the instrument signals of ClNO_2_ observed during the field study. Evidently, I^35^ClNO_2_^−^ (208 amu) was significantly correlated with I^37^ClNO_2_^−^ (210 amu), with a slope of 0.32. This slope was consistent with the natural isotopic ratio of ^37^Cl to ^35^Cl, confirming the identity of the observed ClNO_2_ (*56*). δ^37^Cl is empirically defined as follows

$$\delta^{37}\mathrm{Cl}=\left(\frac{R_{\text{sample}}}{R_{\text{standard}}}-1\right)\times 1000$$ (3)

where *R* represents the isotopic ratio of ^37^Cl to ^35^Cl, with a SMOC (ISL354 NaCl) value of 0.319393 (*15*). In Hong Kong, the converted δ^37^Cl of ClNO_2_ exhibited significant fluctuations during the observation period (fig. S18). However, when arranging the δ^37^Cl values based on the magnitude of the instrument signal (208 amu; ^35^ClNO_2_) from high to low, a noteworthy pattern emerged: High signal values exhibited isotopic stability, while a wide range of δ^37^Cl fluctuations primarily occurred at low signals. δ^37^Cl was influenced by both the measurement uncertainty of I^−^-HR-ToF-CIMS and variations in the isotopes themselves (e.g., arising from different origins and environmental processes) (*57*). Consequently, we could infer that δ^37^Cl was less susceptible to measurement uncertainty when the instrument signal was high and, conversely, was more affected when the signal value was low (fig. S19).

To obtain reliable δ^37^Cl values for ClNO_2_, we employed the SM-K test to iteratively analyze the δ^37^Cl values in descending order of instrument signal strength (*20*). Figure S18 presents the SM-K results for δ^37^Cl, plotting statistics for both forward series (UF) and backward series (UB) against signal magnitudes. The intersection points of UF and UB denote the initiation of an abrupt change (*58*). Upon comprehensive examination of the entire dataset (*n* = 11,127), the initial abrupt change occurred at data point 2920, although it did not reach a significance level of 0.05. After two iterations, a final significant abrupt change was identified at data point 246 (208 amu: 2767 Hz; ClNO_2_ mixing ratio: 1456 pptv; representing 2.2% of the total dataset), which was further confirmed by a subsequent iteration showing no additional intersection point. This result indicated that the 246 data points, verified by the SM-K test, were essentially unaffected by technical challenges and represented the intrinsic features of δ^37^Cl in ClNO_2_ in Hong Kong. To further assess the robustness of our results, we conducted a sensitivity analysis by constructing five data subsets containing 206, 226, 246, 266, and 286 observations, respectively, in 20-point increments. Statistical analyses revealed that the distributions and median values of δ^37^Cl-ClNO_2_ across these subsets were consistent, with no significant differences detected (*P* > 0.05; fig. S20). Each subset encompassed both marine and continental air masses identified in the Hong Kong region (Fig. 2A, air masses A and C), which consistently exhibited distinct isotopic signatures. These results confirm that the application of the SM-K test did not influence the principal conclusions of this study. This method was also successfully applied to extract δ^37^Cl-ClNO_2_ values from measurement data collected in Qingdao, Wangdu, Xi’an, and the chamber experiments. The corresponding data points (462, 168, 171, and 159), accounting for 5.9, 2.5, 12.8, and 5.3% of the total data, yielded minimum ClNO_2_ concentration thresholds of 1447, 946, 638, and 1272 pptv, respectively.

### Estimation of the chlorine isotope fractionation

In the open ocean, the δ^37^Cl-Cl^−^ range of oceanic aerosol has been reported to be +0.4 to +2.5‰ (*19*), which is a result of fresh sea salt reacting with acidic gases (i.e., HNO_3_ and H_2_SO_4_) or N_2_O_5_ and HO*_x_* (*2*, *16*). This range reflects Rayleigh fractionation, where δ^37^Cl varies with *f*_c_, the fraction of chlorine remaining in the condensed phase

$$\delta^{37}{\mathrm{Cl}}_{\text{aerosol}}=\left(\alpha -1\right)\times \text{ln}\left(f_{c}\right)\times 1000$$ (4)

where α is the isotopic fractionation factor in the reactions of fresh sea salt with acidic gases or N_2_O_5_ and has a value of 0.9972 (*16*, *25*). Guided by the principle of mass balance in reactions (e.g., NaCl + HNO_3_ and H_2_SO_4_ → aerosol_NaNO3 and Na2SO4_ + HCl; NaCl + N_2_O_5_ → aerosol_NaNO3_ + ClNO_2_), the following relationship is established

$$\delta^{37}{\mathrm{Cl}}_{\text{NaCl}}=\delta^{37}{\mathrm{Cl}}_{\text{aerosol}}\times f_{c}+\delta^{37}{\mathrm{Cl}}_{\text{HCl or ClNO}2}\times \left(1-f_{c}\right)$$ (5)

where δ^37^Cl_NaCl_ in the ocean has a uniform distribution, nearing 0‰ (*24*). Therefore, δ^37^Cl_HCl or ClNO2_ can be estimated by the formula with *f*_c_

$$\delta^{37}{\mathrm{Cl}}_{\text{HCl or ClNO}2}=\left(\frac{f_{c}}{f_{c}-1}\right)\times \left(\alpha -1\right)\times \text{ln}\left(f_{c}\right)\times 1000$$ (6)

The reaction rate of NaCl with HNO_3_ and H_2_SO_4_ is much greater than that with N_2_O_5_ (*59*), indicating the dominance of reactions with HNO_3_ and H_2_SO_4_ in the atmosphere. By neglecting the N_2_O_5_ reaction, we estimated that the range of oceanic δ^37^Cl-HCl was −1.8 ± 0.7‰; this range precisely aligns with the observed δ^37^Cl-HCl range (−3.5 to −1.2‰) in rainwater (*17*).

The reaction rate of N_2_O_5_ is much lower than that of the acidic gases. Hence, when both acidic gases and N_2_O_5_ coexist, the isotope fractionation in ClNO_2_ formation due to competition is expected to be much greater than that estimated for HCl. The wider range of oceanic δ^37^Cl-ClNO_2_ values observed in our study supports this reasoning. On the basis of the empirical formula of isotope fractionation (*31*), we established the following equation for ClNO_2_

$$\delta^{37}{\mathrm{Cl}}_{\text{ClNO}2}=\sqrt{\frac{\gamma_{1}}{\gamma_{2}}}\times \left(\frac{f_{c}}{f_{c}-1}\right)\times \left(\alpha -1\right)\times \text{ln}\left(f_{c}\right)\times 1000$$ (7)

where γ_1_ and γ_2_ represent the reaction probabilities of NaCl with acidic gases (1 × 10^−2^ at 296 K) and NaCl with N_2_O_5_ (4.5 × 10^−4^ at 296 K), respectively (*59*). Consequently, the oceanic δ^37^Cl-ClNO_2_ values range from −15 to −1‰, with the *f*_c_ values varying from 0.01 to 0.99. The estimation aligns with the characteristics of oceanic δ^37^Cl-ClNO_2_ observed in laboratory simulations (−19 to 0‰) and field observations (−12 to −1‰). The negative value of oceanic δ^37^Cl-ClNO_2_ is due to isotope fractionation and indicates that the degree of isotope fractionation in the conversion of Cl^−^ to ClNO_2_ corresponds to the magnitude of the oceanic δ^37^Cl-ClNO_2_, demonstrating a significant nonlinear relationship with *f*_c_ (*16*). In addition, the calculation of *f*_c_ here follows the expression *f*_c_ = [Cl^−^]/([Cl^−^] + [ClNO_2_]), which assumes a closed system in which Cl^−^ reacts exclusively with N_2_O_5_. In reality, however, the atmosphere is an open and dynamic system where Cl^−^ can simultaneously participate in multiple reactions (*6*). This simplification may therefore introduce some uncertainty into the estimated isotope fractionation. Nonetheless, such an approach has been widely used in isotope geochemistry (*16*, *19*). To minimize this uncertainty, we extend the conventional single-reaction framework by incorporating additional pathways, most notably the Cl^−^–HCl reaction, and introducing a competing-reaction correction factor. This refinement enhances both the accuracy and applicability of isotope fractionation estimates under realistic atmospheric conditions.

### Uncertainty evaluation of the isotopic method using I^−^-HR-ToF-CIMS

To evaluate the uncertainty of our isotopic method, we considered three primary sources: (i) measurement uncertainty associated with the reference material, (ii) absolute error from mass spectrometric detection of isotope signals, and (iii) measurement uncertainty from varying environmental factors during sampling period (*56*). In the absence of a direct ClNO_2_ standard gas, we estimated the isotopic composition of ClNO_2_ produced from seawater, a well-characterized reference material (e.g., SMOC; ISL354 NaCl with a ^37^Cl/^35^Cl ratio of 0.319393) (*24*), using isotope fractionation principles (Eq. 7) (*16*). The theoretical δ^37^Cl-ClNO_2_ value (−10 ± 4‰, *n* = 99) closely aligns with our chamber experiments results for ClNO_2_ derived from seawater (−9 ± 4‰, *n* = 159) based on data processed at 5-min intervals (fig. S21). The combined uncertainty from measuring the reference material was determined to be approximately 1‰ calculated using the standard error of the mean (*60*). For absolute error from mass spectrometric detection, we initially considered two factors: (i) peak identification in the mass spectrum and (ii) signal variation during calibration. On the basis of these, we estimated the relative uncertainties of I^35^ClNO_2_^−^ (*u*_1_) and I^37^ClNO_2_^−^ (*u*_2_) as 1.6% and 1.5%, respectively, giving an inferred relative uncertainty of 2.2% for the ratio *u*_2_/*u*_1_. However, this calculation assumes that *u*_1_ and *u*_2_ are completely independent. In practice, *u*_1_ and *u*_2_ exhibit significant covariance (*P* < 0.01, Student’s *t* test), sharing similar noise sources and variation patterns (*56*). This correlation reduces their independence, and the assumption of independence therefore overestimates the true uncertainty. To address this, we directly calculated uncertainty from the SD of the measured *u*_2_/*u*_1_ ratios, as appropriate for isotope analysis. This approach yielded relative uncertainties of 0.31% for Hong Kong, and 0.15, 0.27, and 0.15 for Qingdao, Wangdu, and Xi’an, respectively. The corresponding δ^37^Cl uncertainties were 3‰, 2‰, 3‰, and 2‰. To assess the influence of environmental factors on measurement uncertainty during sampling, we used stable Cl_2_ as a proxy for ClNO_2_, given their similar properties (*35*). The δ^37^Cl values of Cl_2_ released from chlorine permeation tubes used for calibration were measured under varying temperature and relative humidity during field campaigns, as well as in a 24-hour controlled experiment under ambient conditions. Field data, such as the Qingdao observations (fig. S22), showed no significant difference in δ^37^Cl-Cl_2_ between calibrations (*P* > 0.05; Student’s *t* test), indicating that CIMS measurements are stable across a range of temperature and humidity conditions. The δ^37^Cl-Cl_2_ values remained consistent over the 24-hour measurement period, with a mean of −15‰ and SD of 2‰ (fig. S22), closely matching the variability observed during field calibrations (−16 ± 1‰). These results demonstrate good repeatability and suggest that the isotopic composition of Cl_2_ is not significantly affected by minor fluctuations in environmental conditions, particularly in stable environments such as those maintained by air conditioning. On the basis of these findings, we infer that ClNO_2_ likely exhibits similar stability under typical field conditions. We therefore adopt the observed 2‰ variability in Cl_2_ as a reasonable estimate of the uncertainty associated with environmental factors during ClNO_2_ sampling. Incorporating this component into our uncertainty analysis, the total uncertainty for Hong Kong data was calculated as 4‰. Using the same approach, the total uncertainties for other sites were: Qingdao (3‰), Wangdu (4‰), and Xi’an (3‰). These values are comparable to the SD observed in our chamber experiments for seawater-derived ClNO_2_ (−9 ± 4‰). Although this is not a conventional approach to estimating uncertainty, it provides a practical alternative in the absence of direct ClNO_2_ standards.

Although the uncertainty of this method (~4‰) is higher than that of established chlorine isotope techniques such as TIMS, which typically achieves uncertainties below 1‰ (*15*), an uncertainty of 4‰ is considered reasonable, particularly in the context of chlorine isotope analysis for atmospheric reactive species, which remains in its early stages of methodological development. For example, recently developed methods for measuring organochlorines using gas chromatography–quadrupole mass spectrometry (GC-qMS) also exhibit uncertainties exceeding 2‰ (*53*). Moreover, this level of uncertainty does not compromise the reliability of source attribution, especially in cases where large isotopic contrasts exist (e.g., oceanic versus anthropogenic sources, with differences of ~29‰). Together, our findings demonstrate the reliability of the chlorine isotope method in investigating the geochemical cycling of atmospheric chlorine. Continued advancements in CIMS are also expected to further improve measurement precision and reduce overall uncertainty. Noted, given the method’s uncertainty of approximately 4‰, all δ^37^Cl values measured by this method in this study are retained to single digits for consistency.

### Lagrangian particle dispersion modeling

LPDM was performed using the Hybrid Single-Particle Lagrangian Integrated Trajectory (HYSPLIT) model, driven by the Global Data Assimilation System (GDAS), to identify potential sources of air masses during the events of interest (*23*). The model calculates the particle positions using mean wind and a turbulence transport component after their release from a source point for forward simulations or a receptor for backward runs. In this study, we conducted LPDM analysis for each sampling location: Xi’an, with a 48-hour backward trajectory; Wangdu, with 48-hour and 120-hour backward trajectories; Qingdao, with 48-hour forward and backward trajectories; and Hong Kong, with a 48-hour backward trajectory. In each case, 3000 particles were released every hour over the receptor station. Particle residence times below 100 m were used to identify the “footprint” of air masses arriving at the receptor station. The spatiotemporal distributions of these particles were used to identify the potential source regions and their relative contributions to air masses at specific locations (*61*).

### Bayesian model simulations

The Bayesian mixing model in R (MixSIAR) uses isotopic data to quantify the contributions of individual sources in a mixture, while also accommodating uncertainties related to multiple sources, fractionation, and isotopic signatures (*62*). This approach has been widely adopted for isotopic source apportionment (*19*, *27*). Detailed information regarding the model, available as an open-source R package, is provided in prior research (*19*, *51*). In brief, the model uses isotopic values from various sources as endmembers and uses finite mixing simulation to determine the posterior probability of contribution from each source based on the isotopic values of the samples.

In our study, the Bayesian simulation considered two sources of ClNO_2_: those originated from continental Cl^−^ (+23 ± 7‰) and oceanic Cl^−^ (0 ± 1‰). In addition, since the measured δ^37^Cl-ClNO_2_ did not directly reflect the initial δ^37^Cl-Cl^−^ due to isotope fractionation, a prior calculation of isotope fractionation was conducted, as described above. Notably, within this isotopic quantification framework, ClNO_2_ analysis reflected the contributions of the continental and oceanic sources to its Cl^−^ precursor. Consequently, these quantitative findings were equally relevant to those of Cl^−^ and HCl, elucidating the contribution dynamics of these three chlorine compounds.

### WRF-Chem simulations

In this study, we used the WRF-Chem model with the latest reactive chlorine chemistry scheme to understand the penetration extent of oceanic chlorine on the continents. Hence, we modeled a scenario by excluding anthropogenic chlorine sources and focusing solely on oceanic sources of chlorine (*38*). The simulation spanned from 1 January to 28 February 2023, with the initial month’s data discarded as spin-up. Our simulation domain consisted of the site of interest (Wangdu, indicated by the purple dot in fig. S10). To monitor the temporal variation in the abundance of gaseous reactive chlorine within our domain resulting from oceanic chloride emissions, we set the initial and boundary conditions of all chlorine species to zero. Our setup for the emission inventory of routine air pollutants, chemical mechanisms, and physical schemes followed our previous WRF-Chem simulations, as summarized in the table S2.

## Supplementary Materials

### The PDF file includes:

Supplementary Text S1 to S3 Figs. S1 to S22 Tables S1 and S2 Legend for data S1 References
