# Supplementary material for: Isotopic constraints on the origin of reactive chlorine in the troposphere
Source: Sci Adv. 2026 Jan 16;12(3):eaeb5397. doi: 10.1126/sciadv.aeb5397 (PMC12810628; doi:10.1126/sciadv.aeb5397)
Supplement: Supplementary file 1 — Supplementary Text S1 to S3 Figs. S1 to S22 Tables S1 and S2 Legend for data S1 References [file sciadv.aeb5397_sm.pdf]

Science Advances
AAAS

Supplementary Materials for

Isotopic constraints on the origin of reactive chlorine in the troposphere

Zheng Zong *et al.*

Corresponding author: Likun Xue, xuelikun@sdu.edu.cn; Ru-Jin Huang, rujin.huang@ieecas.cn;
Tao Wang, tao.wang@polyu.edu.hk

Sci. Adv. **12**, eaeb5397 (2026)
DOI: 10.1126/sciadv.aeb5397

The PDF file includes:

Supplementary Text S1 to S3
Figs. S1 to S22
Tables S1 and S2
Legend for data S1
References

Other Supplementary Material for this manuscript includes the following:

Data S1

Text S1. Isotopic evidence for the influence of oceanic sources at Wangdu

To gain a deeper understanding of the extensive transport of oceanic chlorine to the continent, we conducted a detailed analysis of Lagrange Particle Dispersion Model (LPDM) back trajectories coupled with hourly chlorine isotope composition of nitryl chloride (δ^{37}Cl-$ClNO_2$) at Wangdu, as shown in Fig. S9 (*23*). Notably, upon scrutinizing the 48-hour trajectories at 1:00 on February 18, 2023 (local time) and 2:00 ~ 3:00 on February 23, 2023, it is evident that they originated from continental regions. However, when extending the analysis to 120 hours, these air masses had apparently traversed the sea, indicating the infiltration of oceanic sources. Such trajectory patterns align well with their δ^{37}Cl values ($ClNO_2$: +0 ± 1‰, Cl^-: +12 ± 2‰), which were significantly lower than those observed for trajectories that cease to traverse the ocean (δ^{37}Cl-$ClNO_2$: +6 ± 3‰, δ^{37}Cl-Cl^-: +18 ± 4‰). This indicates that oceanic chlorine has the capability to undergo extensive transport to inland regions, with atmospheric residence time extending up to several days (*63*). This phenomenon persists even during the cold season when inland winds prevail, resulting in the widespread presence of oceanic chlorine in the continental regions. Direct field evidence supporting this assertion was evident in air mass trajectories of Wangdu that solely originated from the continent. For example, in specific instances such as those recorded on March 5, 2023, at 0:00 and 10:00, as well as at 0:00 on March 6, 2023, oceanic sources accounted for about 20% of observed $ClNO_2$, underscoring the significant role played by oceanic chlorine in these atmospheric processes.

It is noteworthy that several saline lakes are situated to the northwest of Wangdu and Xi'an (Fig. S7). Previous studies have shown that saline lakes may contribute to local atmospheric reactive chlorine (*63, 64*). Additionally, the chlorine isotopic characteristics of chloride in

saline lakes ($\delta^{37}Cl$-Cl^-) may be similar to those from the ocean (*37*), potentially complicating the identification of oceanic sources at these two locations. To discern their influence on the atmospheric chlorine isotopes observed in this study, we compared the $\delta^{37}Cl$-$ClNO_2$ data possibly associated with saline lakes in Xi'an and Wangdu (Fig. S7). Both sites received airflow with trajectory passing through the saline lake areas during the sampling periods (*45*). However, analysis of the $\delta^{37}Cl$-$ClNO_2$ values in Xi'an indicates no significant difference between trajectories passing through saline lake areas and those bypassing them ($p > 0.05$, Student's t-test). Moreover, the $\delta^{37}Cl$-$ClNO_2$ value at Xi'an was much higher than that at Wangdu, even when passing through saline lake areas (*37*). This suggests that saline lake chlorine has a negligible impact on the atmospheric reactive chlorine at these sites. The primary difference is due to significant influence of oceanic chlorine on Wangdu. The limited chlorine supply from saline lakes and the considerable distance (exceeding 500 km) likely explains their minimal impact on these locations.

Text S2. Introduction of the field measurement campaigns

In this study, field campaigns were conducted at four locations with high atmospheric chlorine activity: Wangdu in North China, Qingdao in East China, Hong Kong in South China, and Xi'an in West China (Fig. 1a). These four stations have been extensively deployed to investigate regional air pollution and atmospheric chemistry processes in China, and their detailed descriptions can be found elsewhere and are provided in the main text (*46, 57, 65*).

$ClNO_2$ was measured using a high-resolution I^--ToF-CIMS during Wangdu, Qingdao and Hong Kong samplings, while the measurement in Xi'an was conducted using an upgraded version of the instrument (I^--LToF-CIMS). The principles of these two instruments are similar

and can be referred to Lee et al. (2014) (*47*). During the observations in Wangdu, Qingdao and Hong Kong, the ion-molecule reaction (IMR) chamber of I^--HR-ToF-CIMS was maintained at a controlled pressure of 100 mbar. The inlet tube, a 0.75 m long 1/2 inch O.D. PFA tube, was replaced every two days to prevent the generation of $ClNO_2$ and other artifacts inside the tube. The background signal of $ClNO_2$ was determined by automatically injecting zero air into I^--HR-ToF-CIMS every 3 hours to ensure accurate derivation of the $ClNO_2$ signal. To calibrate $ClNO_2$ sensitivity, known concentrations of $ClNO_2$ were injected by blowing sufficient N_2O_5 through wet NaCl slurry in a Teflon tube, assuming unity conversion efficiency from N_2O_5 to $ClNO_2$. The detection limit of $ClNO_2$ was determined by injecting synthetic air (comprising N_2 and O_2) into the instrument and observing the standard deviation in $ClNO_2$ signals in a 5-min duration. Combining with the sensitivity of $ClNO_2$ obtained from calibrations, the detection limit was defined as two times the standard deviation in $ClNO_2$ mixing ratios, which is 0.25 pptv on average. The high mass resolution of I^--HR-ToF-CIMS, approximately 4000, ensured effective exclusion of mass interference of $I^{35}ClNO_2^-$ and $I^{37}ClNO_2^-$ (*66*). The final CIMS data was pre-averaged to 5-minute interval from the original 10-second readings. During the Xi'an sampling campaign, instrument operation, time resolution, and data processing were similar to the above three field observations. However, the IMR chamber pressure was maintained at 200 mbar, and the mass resolution of I^--HR-LToF-CIMS was higher, reaching 10,000 (*67*). These conditions resulted in a lower detection limit for $ClNO_2$, approximately 0.11 pptv.

The uncertainty of measuring $ClNO_2$ concentrations consists of the following five parts during the calibration: 1. The uncertainty in peak identification; 2. The variation in $ClNO_2$ signals; 3. The accuracy of NO_x analyzers which measures the conversion from NO_x to $ClNO_2$;

4. Loss of N_2O_5 on the tubing of the calibration system with unknown amounts of $ClNO_2$ production; 5. The uncertainty of assuming a unity conversion from N_2O_5 to $ClNO_2$ (*57*). The associated uncertainty of each part was estimated at 1%, 1%, 15%, 2%, and 1%, respectively, in Hong Kong observations, which yields a propagated uncertainty of 15.2%. Similarly, the propagated uncertainty in Wangdu, Qingdao, and Xi'an is 15.3%, 15.7%, and 20.0%, respectively.

The Cl^- within $PM_{2.5}$ at Wangdu, Qingdao and Hong Kong was measured using an ion chromatography system equipped with a gas and aerosol collector (GAC-IC). As GAC-IC is a mature commercial instrument, its principle is well documented (*68*). During our samplings, the GAC-IC system was calibrated weekly, with a detection limit of approximately 0.05 $\mu g \cdot m^{-3}$. The GAC-IC data were averaged to a time resolution of 1 hour. For the Xi'an observation, a LToF aerosol mass spectrometer (LToF-AMS) was used to measure Cl^- of $PM_{2.5}$ (*69*). This measurement was conducted concurrently with the I^--HR-LToF-CIMS but was unavailable after March 19, 2022, due to the instrument maintenance. LToF-AMS data was processed using Squirrel and PIKA (version 1.25C), and its time series showed good agreement with bulk $PM_{2.5}$ data from the regulatory monitoring network (*69*), validating the measurement accuracy. The detection limit for Cl^- was determined to be 0.03 $\mu g\ m^{-3}$.

In the chamber study, real-time measurements of $ClNO_2$ and dinitrogen pentoxide (N_2O_5) were conducted using the same I^--HR-ToF-CIMS instrument employed in the field observations. In addition to the $ClNO_2$ (208 a.m.u., $I^{35}ClNO_2^-$, exact mass: m/z 207.8668; 210 a.m.u., $I^{37}ClNO_2^-$, exact mass: m/z 209.8639), N_2O_5 was quantified at 235 a.m.u. ($IN_2O_5^-$, exact mass: m/z 234.8857). The I^--HR-ToF-CIMS underwent calibration using the same

methodology as in the field campaigns, yielding detection limits of 0.25 pptv for $ClNO_2$ and 0.16 pptv for N_2O_5, respectively.

Text S3. Isotopic signature of Cl^- after equilibration with HCl in the atmosphere

The isotopic exchange between Cl^- and HCl in the atmosphere may lead to isotopic equilibrium (*56*). Here, we provide a theoretical estimate of their post-equilibrium isotopic compositions. Based on mass balance, the chlorine isotopic composition of HCl emitted during waste incineration can be approximated by the following equation (*70*):

$$\delta^{37}Cl_{HCl} = \left(\delta^{37}Cl_0 - \delta^{37}Cl_{Cl^-} \times f_c\right) / \left(1 - f_c\right) \qquad (S1)$$

where $\delta^{37}Cl_0$ is the initial chlorine isotopic value, and f_c is the fraction of chlorine retained as particulate Cl^-. Using this approach, the estimated $\delta^{37}Cl$ of HCl averages approximately +3‰. Assuming an idealized urban atmosphere where the HCl-to-Cl^- concentration ratio is 3:1 (*4*), and $\delta^{37}Cl$ values of +3‰ for HCl and +13‰ for Cl^- prior to exchange, the final equilibrium values of both species would depend on the equilibrium isotopic fractionation factor (α_1). While α_1 has not been directly measured in this context, we reference analogous reactions over marine aerosols and adopt a tentative α_1 value of 0.997 (*16*). Under this assumption, the equilibrium $\delta^{37}Cl$ values would be approximately +5‰ for HCl and +8‰ for Cl^-, indicating an isotopic offset between the two species after equilibration.

However, it is important to note that the isotopic offset is sensitive to the value of α_1. As α_1 increases, the offset between HCl and Cl^- decreases, and the $\delta^{37}Cl$ of Cl^- may exceed +8‰. Previous studies have shown that isotopic exchange is influenced by environmental factors such as temperature, relative humidity, and aerosol pH, introducing uncertainty into precise predictions of equilibrium $\delta^{37}Cl$ values under ambient atmospheric conditions (*19, 56*).

Furthermore, once Cl^- is introduced into the atmosphere, it may not only equilibrate with HCl but also participate in heterogeneous reactions, such as with N_2O_5 to form $ClNO_2$ (*1*). In such cases, the system may not reach isotopic equilibrium before Cl^- is consumed, particularly in polluted, fast-reacting urban environments (*3*). Therefore, while an exact quantitative prediction of equilibrium $\delta^{37}Cl$ values for Cl^- and HCl remains uncertain, the overall trend remains robust: Cl^- derived from anthropogenic combustion sources retains a relatively enriched $\delta^{37}Cl$ signature, whether or not isotopic equilibrium with HCl is fully achieved. This characteristic enrichment provides a valuable isotopic tracer to distinguish continental combustion-related Cl^- from marine sources, which are typically associated with lower $\delta^{37}Cl$ values.

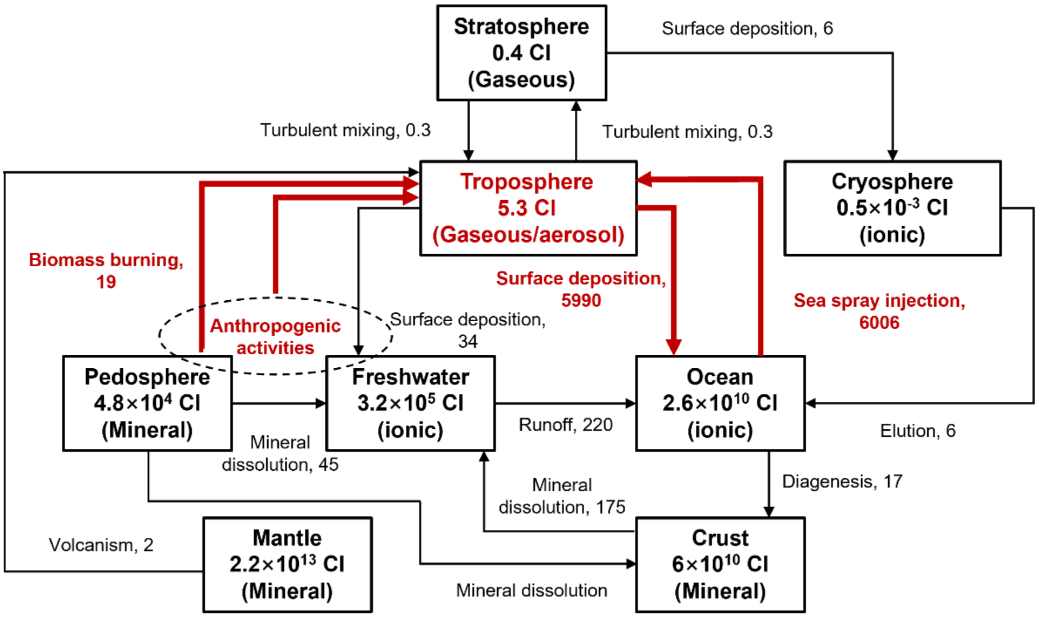

Fig. S1. Budget and cycling of chlorine in the Earth system (*4, 71-74*). Arrows are labeled in fluxes of chlorine in teragram (Tg) per year. The red section, representing the most intense interaction, underscores its critical contribution to tropospheric chlorine.

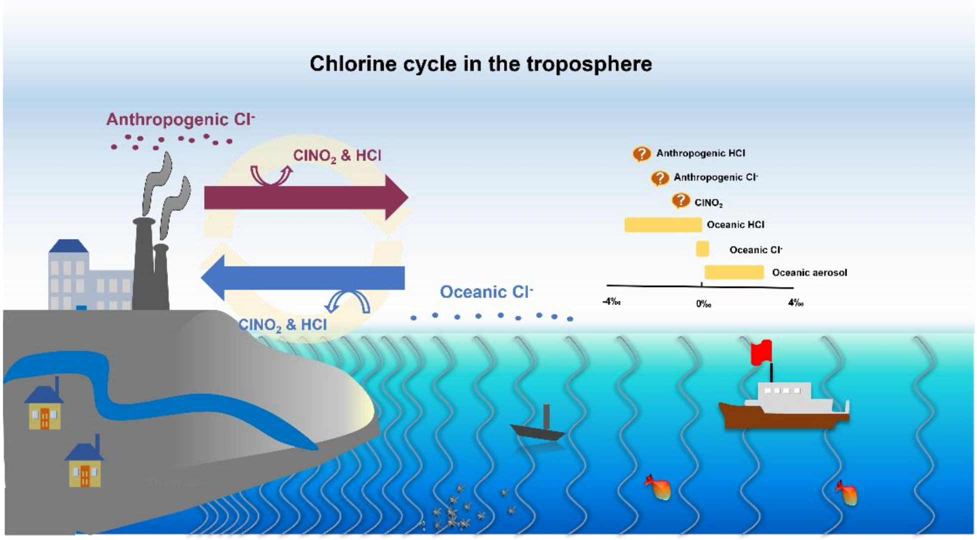

Fig. S2. Geochemical chlorine cycle in the troposphere. The $\delta^{37}Cl$ values for oceanic aerosols, Cl^- and HCl have been reported (*16, 17, 24*), however, data for their anthropogenic counterparts and the pivotal $ClNO_2$ are notably lacking.

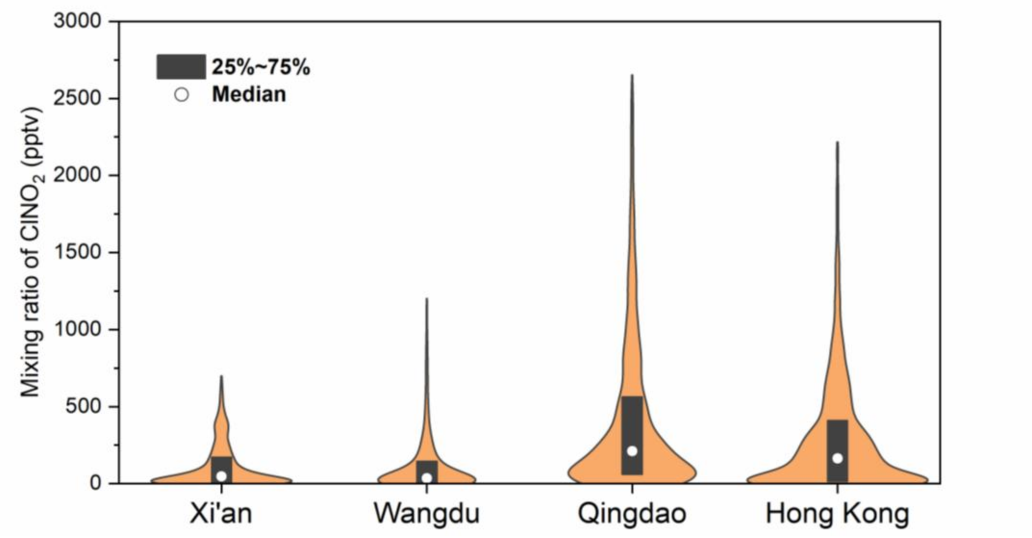

Fig. S3. Distributions of ClNO$_2$ mixing ratios observed in the four regions of China, showing the 5-minute median values along with the 25th to 75th percentile ranges.

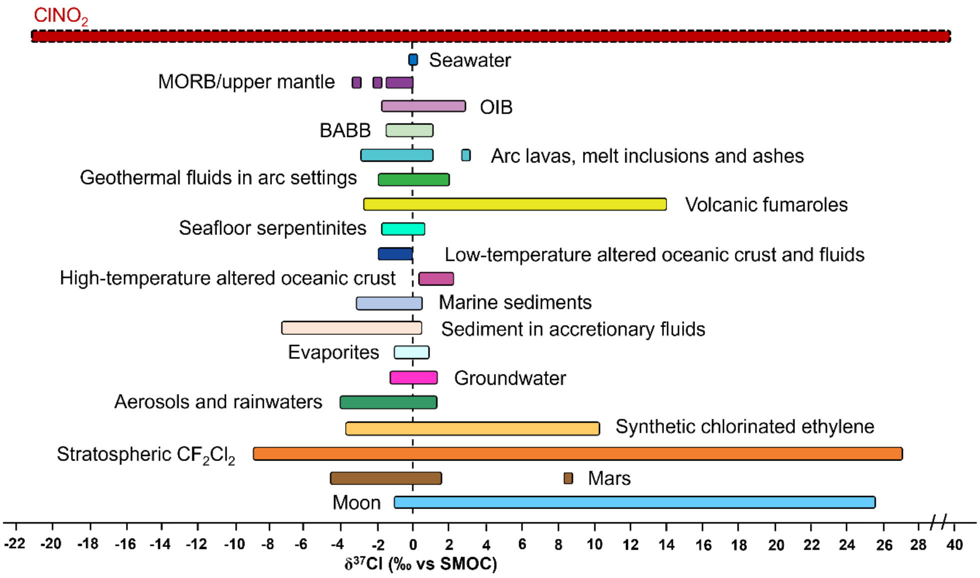

Fig. S4. Reported characteristics of δ^{37}Cl in various reservoirs on Earth and extraterrestrial planets (*54, 75, 76*). The red dashed bar indicates the δ^{37}Cl signature of ClNO$_2$ identified in this study, which significantly exceeds the current knowledge of the δ^{37}Cl range. MORB, OIB and BABB represent mid-ocean ridge basalt, oceanic island basalt, and back-arc basin basalt, respectively.

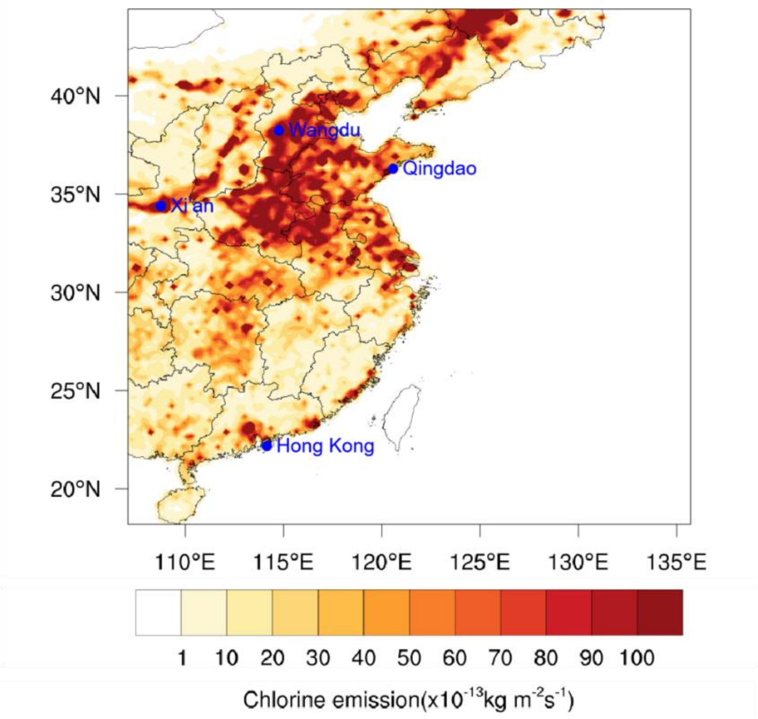

Fig. S5. Monthly average emissions of anthropogenic inorganic chlorine, based on the inventory developed by Fu et al. (2018) (*4*). Specifically, the chlorine emissions include Cl^- and HCl, in units of 10^{-13} kg m^{-2} s^{-1}. Xi'an, Wangdu, and Qingdao are located in regions with comparable levels of anthropogenic chlorine emissions.

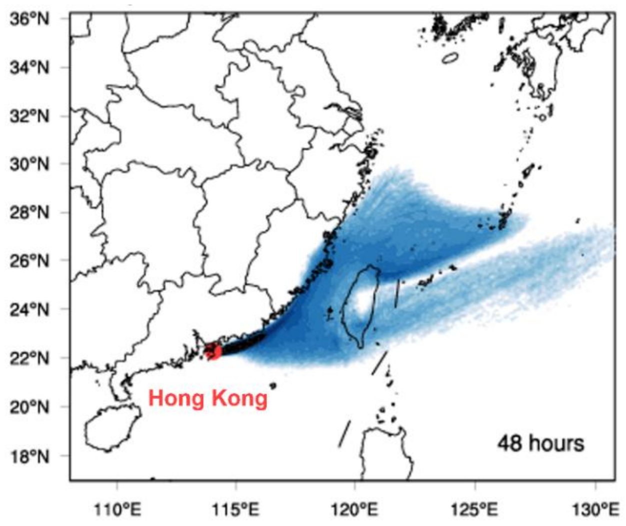

Fig. S6. 48-hour LPDM backward trajectories during the period 2020.11.18 0:00 ~ 06:00 (local time) at Hong Kong. Notably, these air masses were exclusively derived from oceanic regions.

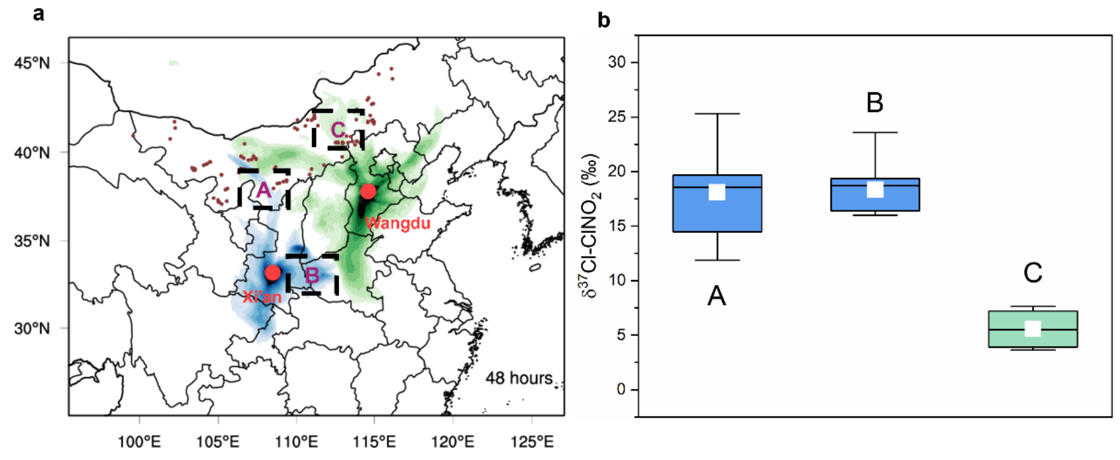

Fig. S7. The 48-hour backward trajectories and hourly δ^{37}Cl-ClNO$_2$ data used to eliminate the impact of saline lakes at Wangdu and Xi'an. (a) Backward air mass trajectories computed by the LPDM model for Xi'an (light blue) and Wangdu (light green) during the observation periods. Particle residence times below 100 m were used to identify the "footprint" of air masses arriving at the receptor station. Areas A and C, marked with black dashed boxes, indicate regions with saline lakes (brown dots), while area B does not contain saline lakes. **(b)** The characteristics of δ^{37}Cl-ClNO$_2$ values in the air masses passing through areas A, B and C.

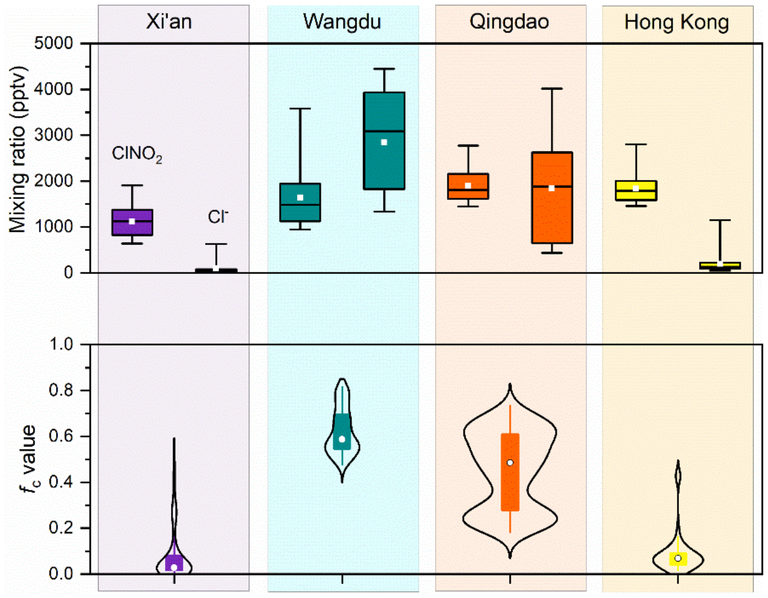

Fig. S8. Characteristics of the $ClNO_2$ and Cl^- concentrations extracted for isotope analysis at the four locations. The parameter f_c denotes the ratio of unreacted Cl^- remaining during its reaction with N_2O_5 to form $ClNO_2$; this is calculated as the mole ratio of Cl^- to the sum of $ClNO_2$ and Cl^- after the reaction of Cl^- with N_2O_5.

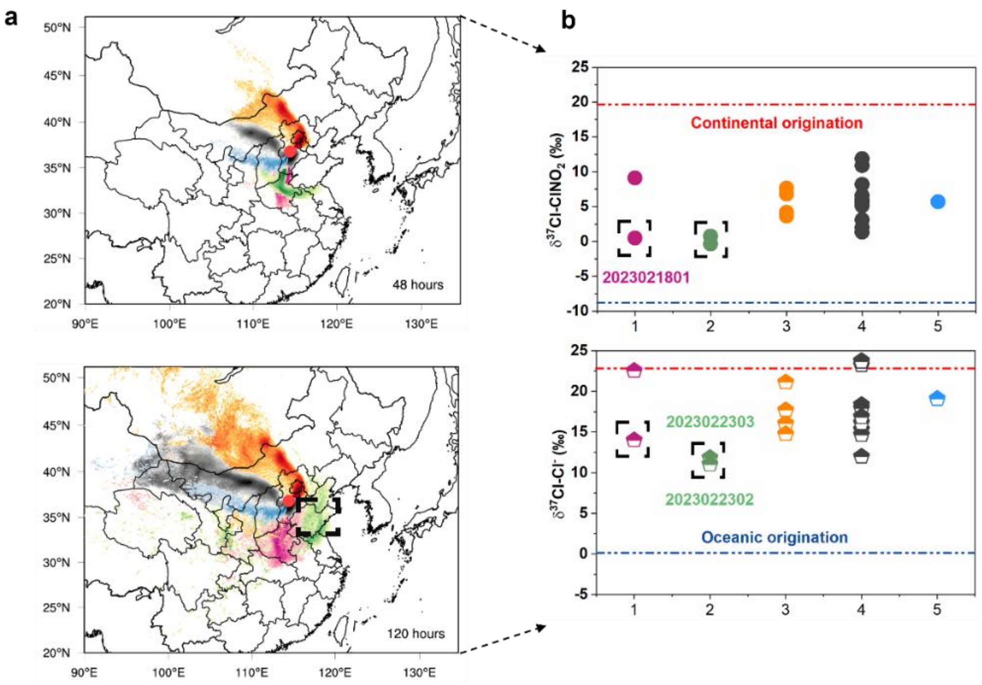

Fig. S9. LPDM back trajectories coupled with hourly δ^{37}Cl-$ClNO_2$ data at Wangdu. (a) 48-hour (upper) and 120-hour (lower) back trajectories for the timestamps 2023021801 and 2023021812 (pink), 2023022302-2023022303 (green), 2023022406-2023022409 (orange), 2023030500-2023030510 (grey), and 2023030600 (blue) (local time: Year/Month/Day/Hour) derived from LPDM simulations. The 120-hour backward trajectories for timestamps 2023021801 and 2023022302-2023022303 show that their air masses passed over the ocean, highlighted with black dashed boxes. **(b)** Corresponding δ^{37}Cl values of $ClNO_2$ and Cl^- in the backward air masses colour coded consistently with panel (a). Backward trajectories clearly exhibit oceanic chlorine signals, indicating the widespread presence of oceanic chlorine in continental regions, even during the cold season when inland winds prevail. Additionally, trajectories that traverse the sea exhibit lower δ^{37}Cl values than those that do not transverse the sea.

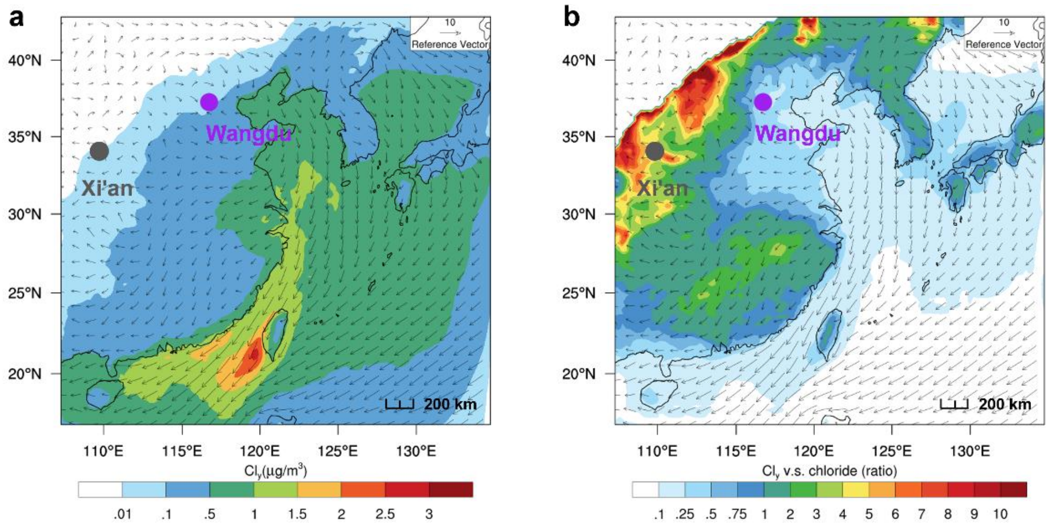

Fig. S10. The sensitivity case simulation results, using a WRF-Chem model with the latest reactive chlorine chemistry, considering only oceanic sources of reactive chlorine, excluding anthropogenic sources. (a) The color scale represents the average concentration of gaseous chlorine (Cl_y) in February 2023, with a purple dot and a gray dot marking the Wangdu and Xi'an locations, respectively. **(b)** The color scale depicts the ratio of Cl_y to Cl^-, indicating that gaseous chlorine from the ocean becomes increasingly dominant as it penetrates inland.

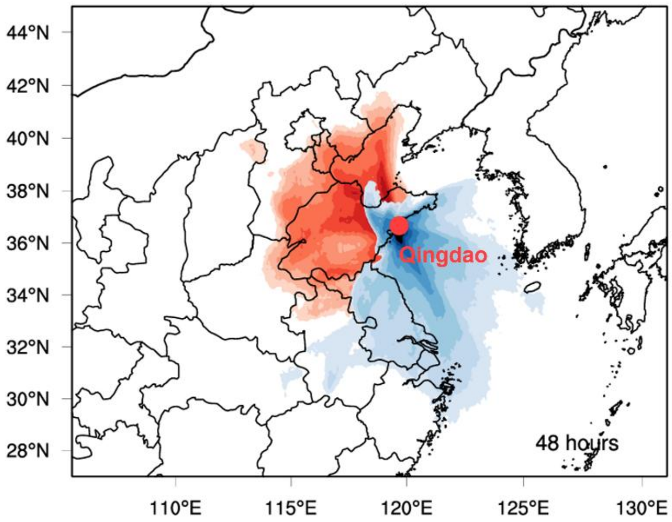

Fig. S11. Characteristics of 48-hour air mass trajectories computed by LPDM model in Qingdao during the observation period. Red trajectories represent backward air masses, while blue trajectories represent forward air masses. The forward trajectories illustrate the transport of anthropogenic chlorine to the ocean, impacting offshore air quality.

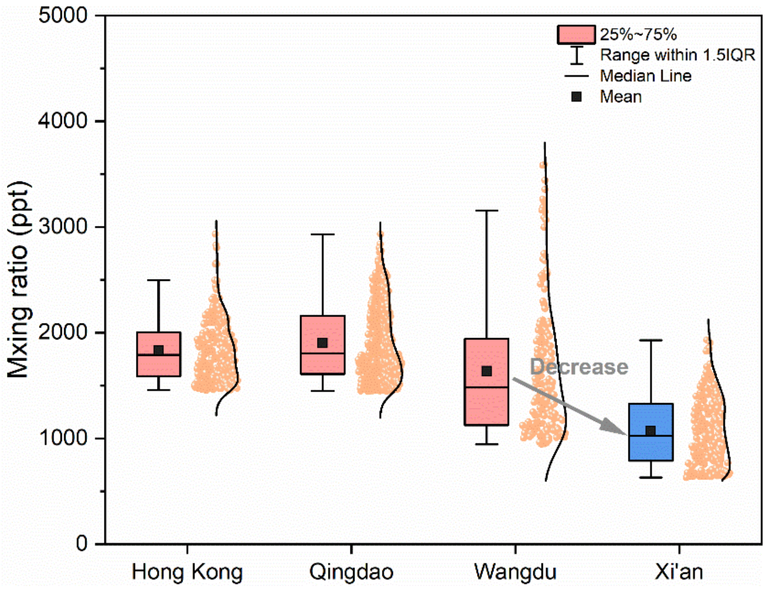

Fig. S12. Mixing ratio distributions of $ClNO_2$ extracted for isotope analysis at the four sampling

sites. Observations in Hong Kong, Qingdao and Wangdu were conducted using I^--HR-ToF-CIMS, while the Xi'an observation utilized I^--HR-LToF-CIMS. It is obvious that isotope detection limit of I^--HR-LToF-CIMS is 1.5 times lower than that of I^--HR-ToF-CIMS. This improvement suggests that with continued technical advancements, it will become possible to detect other reactive chlorine species present in trace amounts, such as Cl_2.

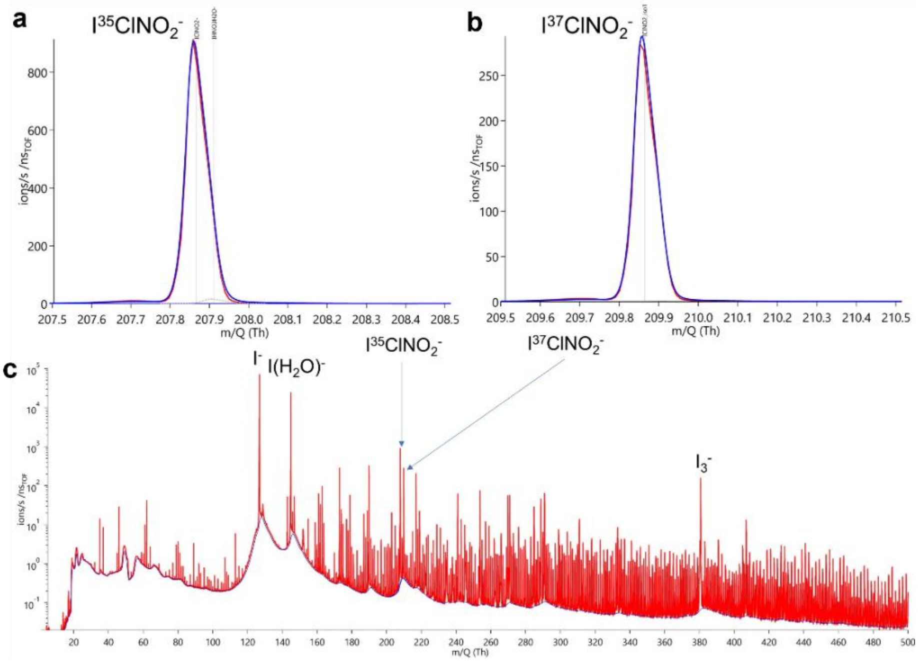

Fig. S13. Detection of $I^{35}ClNO_2^-$ and $I^{37}ClNO_2^-$ using I^--HR-ToF-CIMS, exemplified by data signals from Hong Kong. (a) High-resolution peak fitting of $I^{35}ClNO_2^-$ at 208 m/Q. **(b)** High-resolution peak fitting of $I^{37}ClNO_2^-$ at 210 m/Q. **(c)** Mass spectrum from 10 to 500 m/Q, showing the position of the reagent ions (I^- and IH_2O^-) and target ions ($I^{35}ClNO_2^-$ and $I^{37}ClNO_2^-$).

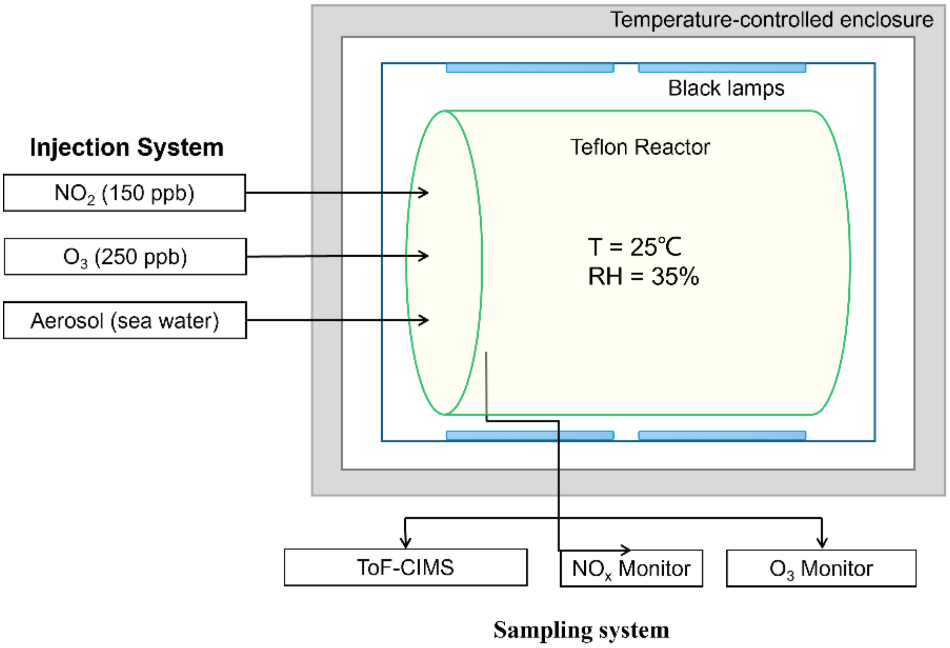

Fig. S14. Schematic diagram illustrating the laboratory simulation of oceanic $\delta^{37}Cl$-$ClNO_2$. $ClNO_2$ is produced through the reaction between fresh sea salt particles and N_2O_5.

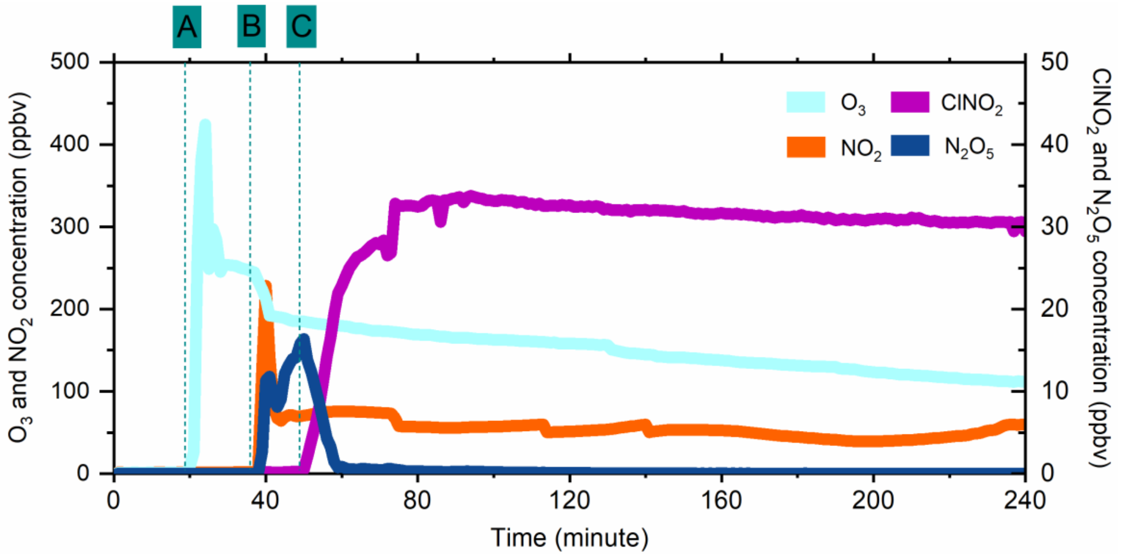

Fig. S15. Experimental process for producing oceanic $ClNO_2$; this involves the reaction of fresh sea salt with N_2O_5 in a smog chamber. Points A, B and C indicate the times at which O_3, NO_2, and fresh sea salt particles were injected into the chamber, respectively. The concentrations of O_3 and NO_2 are shown on the left Y-axis, while those of $ClNO_2$ and N_2O_5 are displayed on the right Y-axis.

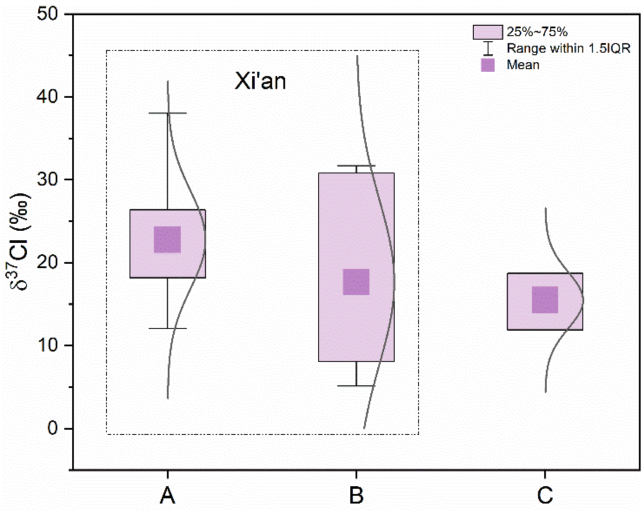

Fig. S16. Comparative analysis of δ^{37}Cl-Cl$^-$ signatures from continental anthropogenic sources. A is the estimated δ^{37}Cl values based on $ClNO_2$ isotopic signatures observed in Xi'an; B refers to the isotope signature of fly ash from waste incineration in Xi'an, analyzed via conventional thermal ionization mass spectrometry (TIMS) (*34*); C is the isotope characteristics of fly ash from waste incineration in Shaoxing, Zhejiang Province, assessed using traditional TIMS.

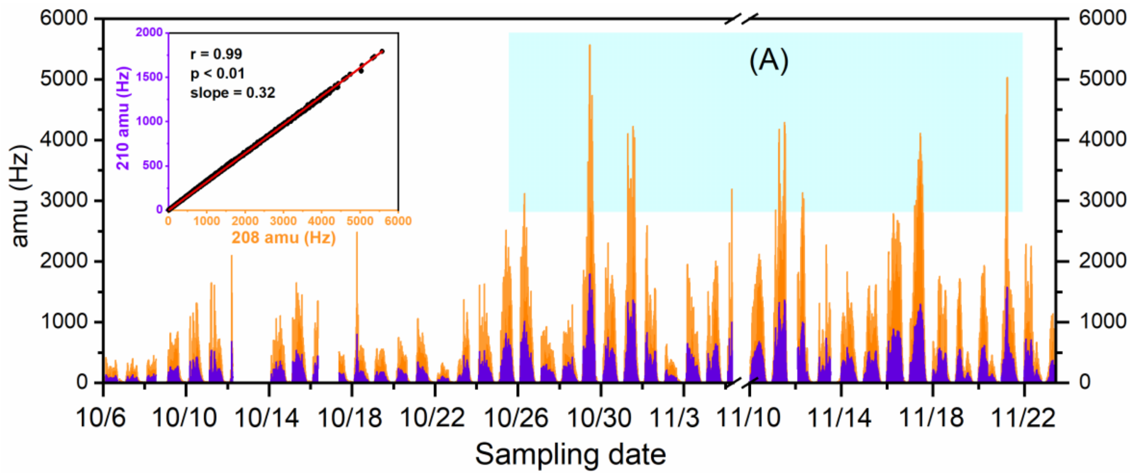

Fig. S17. Time series of the I$^-$-HR-Tof-CIMS signals of $ClNO_2$ during the Hong Kong observational campaign. Orange represents the signal at 208 a.m.u. ($I^{35}ClNO_2^-$), while purple represents the signal at 210 a.m.u. ($I^{37}ClNO_2^-$). These data within the cyan area (A) meet the criteria for isotopic analysis.

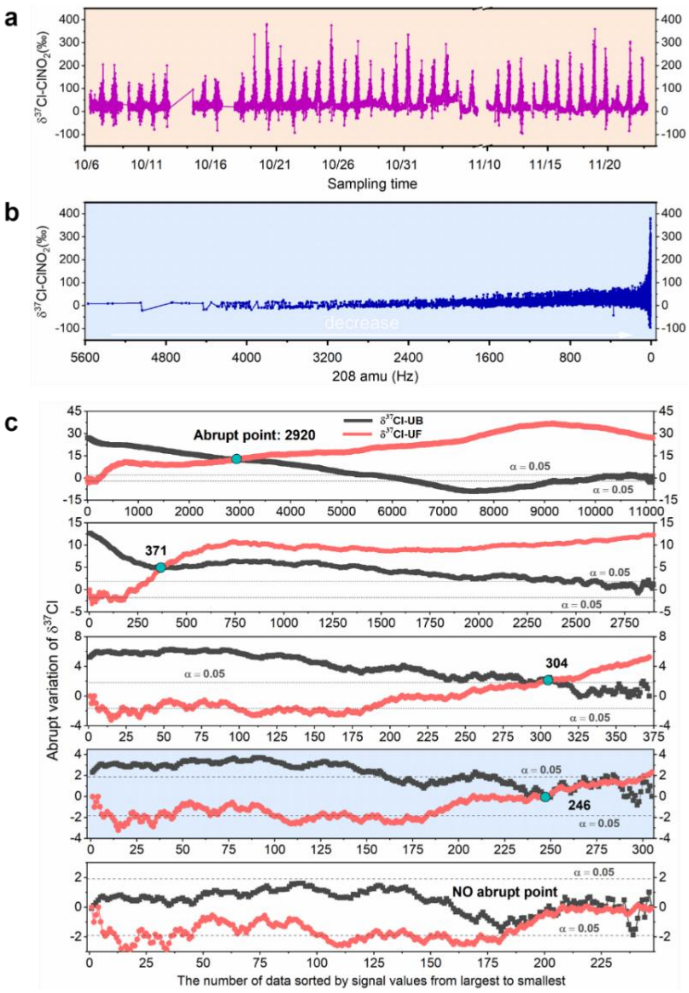

Fig. S18. Specific process for extracting the effective $ClNO_2$ chlorine isotope data from Hong Kong observations. (a) Time series of δ^{37}Cl-$ClNO_2$ measurements in Hong Kong, where the original data were averaged into 5-minute intervals, yielding 11127 data points with a maximum signal of 5600 Hz. **(b)** Arrangement of δ^{37}Cl-$ClNO_2$ according to the magnitude of the instrument signal (208 a.m.u.), ordered from high to low. **(c)** Iterative SM-K test processes to extract reliable δ^{37}Cl values of $ClNO_2$, plotting statistics for both the forward series (UF) and backward series (UB) against the magnitude of the signals.

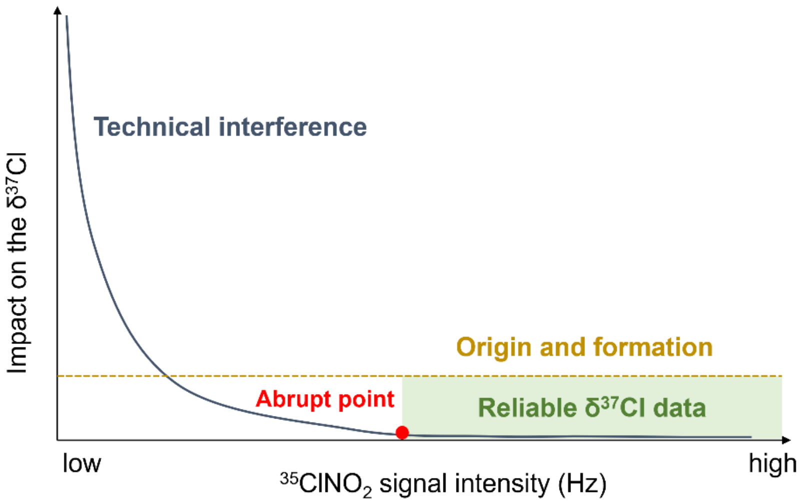

Fig. S19. Conceptual diagram illustrating the extraction of effective δ^{37}Cl-$ClNO_2$ signals using the Sequential Mann-Kendall (SM-K) test (*22*). At a given moment, the isotopic composition of $ClNO_2$, determined by its origin and formation process, remains constant. The influence of technical interference decreases as the $ClNO_2$ signal increases. When the $ClNO_2$ concentration reaches a sufficiently high level, this interference becomes negligible, allowing the true isotopic variation to be accurately observed. This threshold is referred to as the "abrupt point".

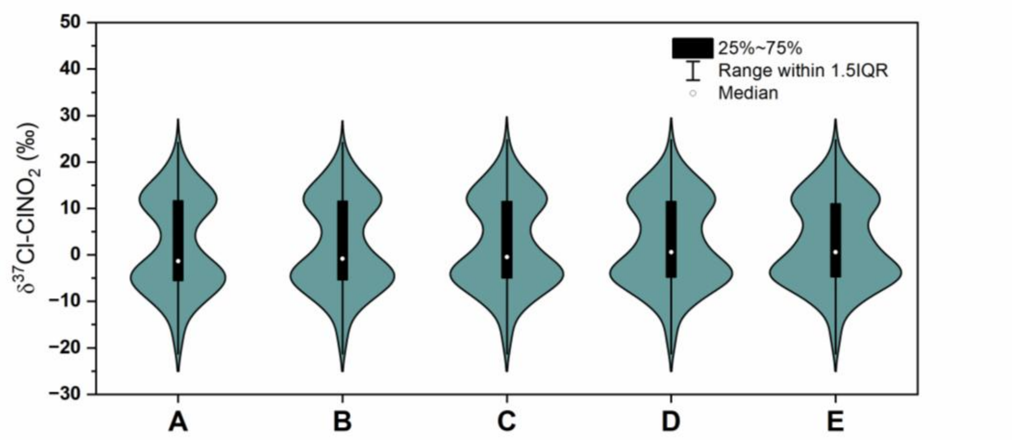

Fig. S20. Distributions of δ^{37}Cl-$ClNO_2$ values for five data subsets consisting of 206 (A), 226 (B), 246 (C), 266 (D), and 286 (E) observations. The subset with 246 data points was selected using the Mann-Kendall (SM-K) test. The distributions show consistent median values and patterns across all subsets.

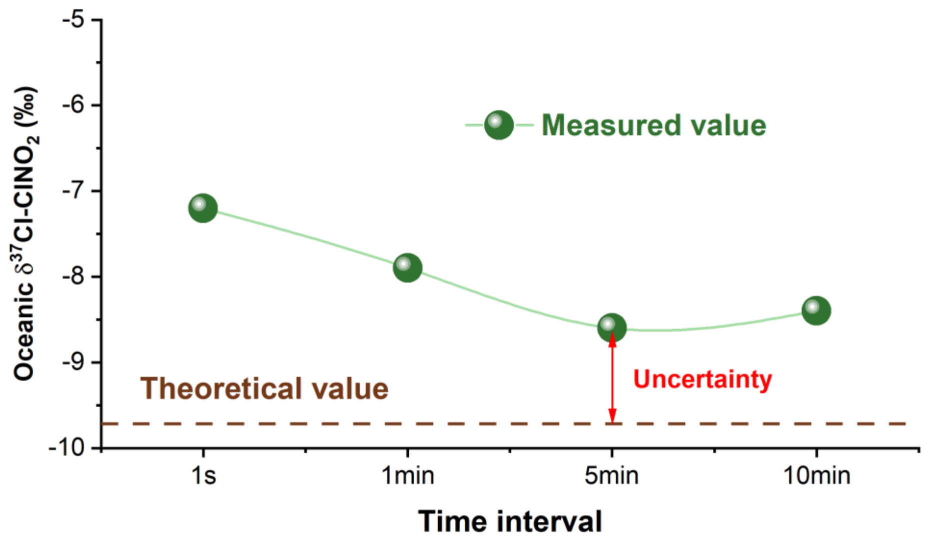

Fig. S21. Comparison of oceanic $ClNO_2$ isotope data between the theoretical value and measured values processed at 1s, 1 min, 5 min, and 10 min intervals using the established method in our chamber study. The 5-min interval data showed the smallest deviation from the theoretical value, indicating that this processing interval is the most suitable for isotopic analysis (*16, 25*).

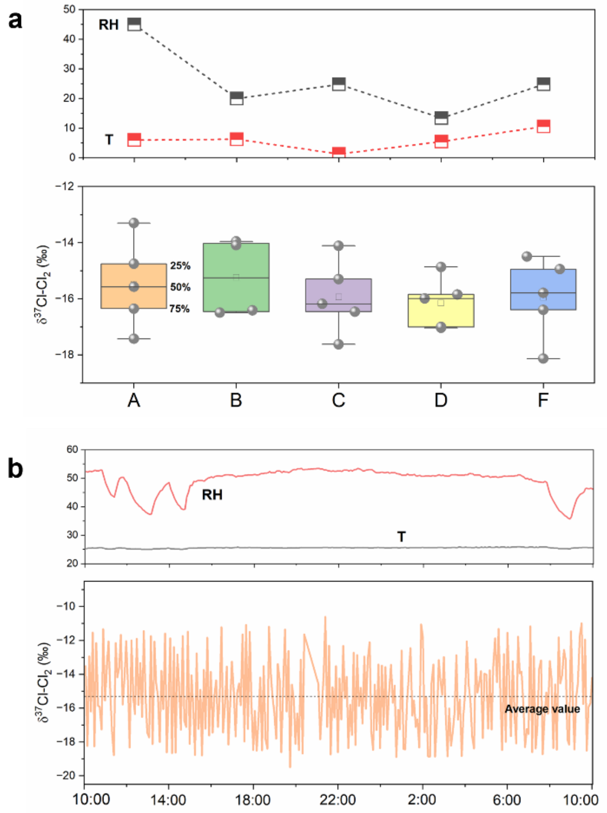

Fig. S22. Isotopic composition (δ^{37}Cl, 5-minute intervals) of Cl_2 released from chlorine permeation tubes. (a) δ^{37}Cl values of Cl_2 from permeation tubes used for calibration during field measurements in Qingdao, shown together with the corresponding relative humidity (RH) and temperature (T) of the diluted gas. Panels A–F correspond to calibrations conducted on February 10, 11, 12, 13, and 22, 2023.

(b) $\delta^{37}Cl$ values of Cl_2 measured over a 24-hour controlled simulation using a chlorine permeation tube, presented alongside the corresponding ambient RH and T conditions.

Table S1. Two primary atmospheric chlorine cycles that prolong the lifetime of Cl^- and its impact on $ClNO_2$ (*42*).

**Cycle1**	**coarse-mode Cl^- → $ClNO_2$ →Cl radical → HCl → fine-mode Cl^- → $ClNO_2$**
	$Cl^- + N_2O_5 = ClNO_2 + NO_3^-$
	$ClNO_2$ + hv = Cl radical + NO_2
	Cl radical + VOCs = RO_2 + HCl
	HCl(g) = HCl(aq), HCl(aq) = $H^+ + Cl^-$
	$Cl^- + N_2O_5 = ClNO_2 + NO_3^-$
**Cycle2**	**coarse-mode Cl^- → HCl → Cl radical → HCl →fine-mode Cl^- → $ClNO_2$**
	NaCl + HNO_3(g) = $NaNO_3$ + HCl(g), $Cl^- + H^+$ = HCl(aq), HCl(aq) = HCl(g)
	HCl + OH radical = H_2O + Cl radical
	Cl radical + VOCs = RO_2 + HCl
	HCl(g) = HCl(aq), HCl(aq) = $H^+ + Cl^-$
	$Cl^- + N_2O_5 = ClNO_2 + NO_3^-$

Table S2. Detailed information about the configuration of the WRF-Chem model. The simulation of sea-salt emissions is based on the parameterization by Gong et al. (2003) (*77*), which describes an exponential relationship between sea-salt emissions and surface wind speeds. For aerosol treatment, a revised version of the four-bin MOSAIC module, with size bins of 0.039–0.156 μm, 0.156–0.625 μm, 0.625–2.5 μm, and 2.5–10.0 μm is used (*38, 78*). The dry deposition of chloride and chlorine-containing species is handled using the Wesely scheme (*79*), while wet deposition follows the methods described by Grell and Dévényi (2002) and Badia et al. (2019) (*80, 81*).

Category	Options
Shortwave radiation	RRTMG shortwave radiation scheme (*82*)
Longwave radiation	RRTMG longwave radiation scheme (*82*)
Land-surface model	Noah land surface model (*83*)
Microphysics scheme	Morrison double-moment scheme (*84*)
PBL scheme	Quasi-Normal Scale Elimination PBL scheme (*85*)
Horizontal resolution	27 km
Gas chemistry	MOZART (*81, 86*)
Aerosol chemistry	MOSAIC (*78, 81*)
Photolysis	FTUV (*78, 87*)
Sea-salt aerosol emissions	Gong, 2003 (*77*)
Air pollutant emissions	www.meicmodel.org

Data S1. The dataset used to generate the main figures of this study.

REFERENCES

1. H. D. Osthoff, J. M. Roberts, A. R. Ravishankara, E. J. Williams, B. M. Lerner, R. Sommariva, T. S. Bates, D. Coffman, P. K. Quinn, J. E. Dibb, H. Stark, J. B. Burkholder, R. K. Talukdar, J. Meagher, F. C. Fehsenfeld, S. S. Brown, High levels of nitryl chloride in the polluted subtropical marine boundary layer. *Nat. Geosci.* **1**, 324–328 (2008).

2. A. Saiz-Lopez, R. P. P. Fernandez, Q. Li, C. A. A. Cuevas, X. Fu, D. E. E. Kinnison, S. Tilmes, A. S. S. Mahajan, J. C. Gomez Martin, F. Iglesias-Suarez, R. Hossaini, J. M. C. Plane, G. Myhre, J.-F. Lamarque, Natural short-lived halogens exert an indirect cooling effect on climate. *Nature* **618**, 967–973 (2023).

3. J. A. Thornton, J. P. Kercher, T. P. Riedel, N. L. Wagner, J. Cozic, J. S. Holloway, W. P. Dube, G. M. Wolfe, P. K. Quinn, A. M. Middlebrook, B. Alexander, S. S. Brown, A large atomic chlorine source inferred from mid-continental reactive nitrogen chemistry. *Nature* **464**, 271–274 (2010).

4. X. Fu, T. Wang, S. Wang, L. Zhang, S. Cai, J. Xing, J. Hao, Anthropogenic emissions of hydrogen chloride and fine particulate chloride in China. *Environ. Sci. Technol.* **52**, 1644–1654 (2018).

5. M. A. K. Khalil, R. M. Moore, D. B. Harper, J. M. Lobert, D. J. Erickson, V. Koropalov, W. T. Sturges, W. C. Keene, Natural emissions of chlorine-containing gases: Reactive chlorine emissions inventory. *J. Geophys. Res. Atmos.* **104**, 8333–8346 (1999).

6. X. Wang, D. J. Jacob, S. D. Eastham, M. P. Sulprizio, L. Zhu, Q. Chen, B. Alexander, T. Sherwen, M. J. Evans, B. H. Lee, J. D. Haskins, F. D. Lopez-Hilfiker, J. A. Thornton, G. L. Huey, H. Liao, The role of chlorine in global tropospheric chemistry. *Atmos. Chem. Phys.* **19**, 3981–4003 (2019).

7. J. Liao, L. G. Huey, Z. Liu, D. J. Tanner, C. A. Cantrell, J. J. Orlando, F. M. Flocke, P. B. Shepson, A. J. Weinheimer, S. R. Hall, K. Ullmann, H. J. Beine, Y. Wang, E. D. Ingall, C. R. Stephens, R. S. Hornbrook, E. C. Apel, D. Riemer, A. Fried, R. L. Mauldin III, J. N. Smith, R. M. Staebler, J. A. Neuman, J. B. Nowak, High levels of molecular chlorine in the Arctic atmosphere. *Nat. Geosci.* **7**, 91–94 (2014).

8. J. Dai, Y. Liu, P. Wang, X. Fu, M. Xia, T. Wang, The impact of sea-salt chloride on ozone through heterogeneous reaction with N_2O_5 in a coastal region of south China. *Atmos. Environ.* **236**, 117604 (2020).

9. X. Yang, T. Wang, M. Xia, X. Gao, Q. Li, N. Zhang, Y. Gao, S. Lee, X. Wang, L. Xue, L. Yang, W. Wang, Abundance and origin of fine particulate chloride in continental China. *Sci. Total Environ.* **624**, 1041–1051 (2018).

10. W. Zhou, J. Zhao, B. Ouyang, A. Mehra, W. Xu, Y. Wang, T. J. Bannan, S. D. Worrall, M. Priestley, A. Bacak, Q. Chen, C. Xie, Q. Wang, J. Wang, W. Du, Y. Zhang, X. Ge, P. Ye, J. D. Lee, P. Fu, Z. Wang, D. Worsnop, R. Jones, C. J. Percival, H. Coe, Y. Sun, Production of N_2O_5 and $ClNO_2$ in summer in urban Beijing, China. *Atmos. Chem. Phys.* **18**, 11581–11597 (2018).

11. W. Wei, M. Kastner, A. Spivack, Chlorine stable isotopes and halogen concentrations in convergent margins with implications for the Cl isotopes cycle in the ocean. *Earth Planet. Sci. Lett.* **266**, 90–104 (2008).

12. C.-Q. Liu, Y.-C. Lang, H. Satake, J. Wu, S.-L. Li, Identification of anthropogenic and natural inputs of sulfate and chloride into the karstic ground water of Guiyang, SW China: Combined $\delta^{37}Cl$ and $\delta^{34}S$ approach. *Environ. Sci. Technol.* **42**, 5421–5427 (2008).

13. Z. D. Sharp, J. D. Barnes, A. J. Brearley, M. Chaussidon, T. P. Fischer, V. S. Kamenetsky, Chlorine isotope homogeneity of the mantle, crust and carbonaceous chondrites. *Nature* **446**, 1062–1065 (2007).

14. R. Kaufmann, A. Long, H. Bentley, S. Davis, Natural chlorine isotope variations. *Nature* **309**, 338–340 (1984).

15. Y. K. Xiao, Y. M. Zhou, Q. Z. Wang, H. Z. Wei, W. G. Liu, C. J. Eastoe, A secondary isotopic reference material of chlorine from selected seawater. *Chem. Geol.* **182**, 655–661 (2002).

16. C. Volpe, A. J. Spivack, Stable chlorine isotopic composition of marine aerosol particles in the western Atlantic Ocean. *Geophys. Res. Lett.* **21**, 1161–1164 (1994).

17. G. Koehler, L. I. Wassenaar, The stable isotopic composition ($^{37}Cl/^{35}Cl$) of dissolved chloride in rainwater. *Appl. Geochem.* **25**, 91–96 (2010).

18. M. Bonifacie, "Chlorine isotopes," in *Encyclopedia of Geochemistry: A Comprehensive Reference Source on the Chemistry of the Earth*, W. M. White Ed., (Springer Cham, 2018), pp. 244–248.

19. Z. Zong, X. Wang, C. Tian, Y. Chen, Y. Fang, F. Zhang, C. Li, J. Sun, J. Li, G. Zhang, First assessment of NO_x sources at a regional background site in North China using isotopic analysis linked with modeling. *Environ. Sci. Technol.* **51**, 5923–5931 (2017).

20. T. Mohsin, W. A. Gough, Trend analysis of long-term temperature time series in the Greater Toronto Area (GTA). *Theor. Appl. Clim.* **101**, 311–327 (2010).

21. M. Xia, X. Peng, W. Wang, C. Yu, Z. Wang, Y. J. Tham, J. Chen, H. Chen, Y. Mu, C. Zhang, P. Liu, L. Xue, X. Wang, J. Gao, H. Li, T. Wang, Winter $ClNO_2$ formation in the region of fresh anthropogenic emissions: Seasonal variability and insights into daytime peaks in northern China. *Atmos. Chem. Phys.* **21**, 15985–16000 (2021).

22. J. C. Laube, J. Kaiser, W. T. Sturges, H. Boenisch, A. Engel, Chlorine isotope fractionation in the stratosphere. *Science* **329**, 1167–1167 (2010).

23. A. F. Stein, R. R. Draxler, G. D. Rolph, B. J. B. Stunder, M. D. Cohen, F. Ngan, NOAA's HYSPLIT atmospheric transport and dispersion modeling system. *Bull. Am. Meteorol. Soc.* **96**, 2059–2077 (2015).

24. P. V. Shirodkar, Y. K. Xiao, A. Sarkar, S. G. Dalal, A. R. Chivas, Influence of air–sea fluxes on chlorine isotopic composition of ocean water: Implications for constancy in $\delta^{37}Cl$—A statistical inference. *Environ. Int.* **32**, 235–239 (2006).

25. J. S. Ray, R. Ramesh, Rayleigh fractionation of stable isotopes from a multicomponent source. *Geochim. Cosmochim. Acta* **64**, 299–306 (2000).

26. C. Dorer, P. Hoehener, N. Hedwig, H.-H. Richnow, C. Vogt, Rayleigh-based concept to tackle strong hydrogen fractionation in dual isotope analysis-the example of ethylbenzene degradation by *Aromatoleum aromaticum*. *Environ. Sci. Technol.* **48**, 5788–5797 (2014).

27. Z. Zong, Y. Tan, X. Wang, C. Tian, J. Li, Y. Fang, Y. Chen, S. Cui, G. Zhang, Dual-modelling-based source apportionment of NO_x in five Chinese megacities: Providing the isotopic footprint from 2013 to 2014. *Environ. Int.* **137**, 105592 (2020).

28. B. Xu, G. Zhang, O. Gustafsson, K. Kawamura, J. Li, A. Andersson, S. Bikkina, B. Kunwar, A. Pokhrel, G. Zhong, S. Zhao, J. Li, C. Huang, Z. Cheng, S. Zhu, P. Peng, G. Sheng, Large contribution of fossil-derived components to aqueous secondary organic aerosols in China. *Nat. Commun.* **13**, 5115 (2022).

29. R.-J. Huang, Y. Zhang, C. Bozzetti, K.-F. Ho, J.-J. Cao, Y. Han, K. R. Daellenbach, J. G. Slowik, S. M. Platt, F. Canonaco, P. Zotter, R. Wolf, S. M. Pieber, E. A. Bruns, M. Crippa, G. Ciarelli, A. Piazzalunga, M. Schwikowski, G. Abbaszade, J. Schnelle-Kreis, R. Zimmermann, Z. An, S. Szidat, U. Baltensperger, I. El Haddad, A. S. H. Prevot, High secondary aerosol contribution to particulate pollution during haze events in China. *Nature* **514**, 218–222 (2014).

30. F. Keppler, J. D. Barnes, A. Horst, E. Bahlmann, J. Luo, T. Nadalig, M. Greule, S. C. Hartmann, S. Vuilleumier, Chlorine isotope fractionation of the major chloromethane degradation processes in the environment. *Environ. Sci. Technol.* **54**, 1634–1645 (2020).

31. E. A. Schauble, G. R. Rossman, H. P. Taylor, Theoretical estimates of equilibrium chlorine-isotope fractionations. *Geochim. Cosmochim. Acta* **67**, 3267–3281 (2003).

32. Y. Pan, S. Tian, D. Liu, Y. Fang, X. Zhu, Q. Zhang, B. Zheng, G. Michalski, Y. Wang, Fossil fuel combustion-related emissions dominate atmospheric ammonia sources during severe haze episodes: Evidence from ^{15}N-stable isotope in size-resolved aerosol ammonium. *Environ. Sci. Technol.* **50**, 8049–8056 (2016).

33. M. Czarnacki, S. Hałas, Isotope fractionation in aqua-gas systems: Cl_2-HCl-Cl^-, Br_2-HBr-Br^- and H_2S-S^{2-}. *Isotopes Environ. Health Stud.* **48**, 55–64 (2012).

34. M. Numata, N. Nakamura, H. Koshikawa, Y. Terashima, Chlorine stable isotope measurements of chlorinated aliphatic hydrocarbons by thermal ionization mass spectrometry. *Anal. Chim. Acta* **455**, 1–9 (2002).

35. X. Peng, T. Wang, W. Wang, A. R. Ravishankara, C. George, M. Xia, M. Cai, Q. Li, C. M. Salvador, C. Lau, X. Lyu, C. N. Poon, A. Mellouki, Y. Mu, M. Hallquist, A. Saiz-Lopez, H. Guo, H. Herrmann, C. Yu, J. Dai, Y. Wang, X. Wang, A. Yu, K. Leung, S. Lee, J. Chen, Photodissociation of particulate nitrate as a source of daytime tropospheric Cl_2. *Nat. Commun.* **13**, 939 (2022).

36. L. Jaegle, P. K. Quinn, T. S. Bates, B. Alexander, J. T. Lin, Global distribution of sea salt aerosols: New constraints from in situ and remote sensing observations. *Atmos. Chem. Phys.* **11**, 3137–3157 (2011).

37. W. G. Liu, Y. K. Xiao, Q. Z. Wang, H. P. Qi, Y. H. Wang, Y. M. Zhou, P. V. Shirodkar, Chlorine isotopic geochemistry of salt lakes in the Qaidam Basin, China. *Chem. Geol.* **136**, 271–279 (1997).

38. Q. Li, A. Badia, T. Wang, G. Sarwar, X. Fu, L. Zhang, Q. Zhang, J. Fung, C. A. Cuevas, S. Wang, B. Zhou, A. Saiz-Lopez, Potential effect of halogens on atmospheric oxidation and air quality in China. *J. Geophys. Res. Atmos.* **125**, e2019JD032058 (2020).

39. R. Hossaini, M. P. Chipperfield, A. Saiz-Lopez, R. Fernandez, S. Monks, W. Feng, P. Brauer, R. von Glasow, A global model of tropospheric chlorine chemistry: Organic versus inorganic sources and impact on methane oxidation. *J. Geophys. Res. Atmos.* **121**, 14271–14297 (2016).

40. J. Dai, T. Wang, H. Shen, M. Xia, W. Sun, P. Guy, Significant impact of a daytime halogen oxidant on coastal air quality. *Environ. Sci. Technol.* **59**, 2169–2180 (2025).

41. Y. Zhang, J. Liu, W. Tao, S. Xiang, H. Liu, K. Yi, H. Yang, J. Xu, Y. Wang, J. Ma, X. Wang, J. Hu, Y. Wan, X. Wang, S. Tao, Impacts of chlorine emissions on secondary pollutants in China. *Atmos. Environ.* **246**, 118177 (2021).

42. X. Zhong, H. Shen, M. Zhao, J. Zhang, Y. Sun, Y. Liu, Y. Zhang, Y. Shan, H. Li, J. Mu, Y. Yang, Y. Nie, J. Tang, C. Dong, X. Wang, Y. Zhu, M. Guo, W. Wang, L. Xue, Nitrous acid budgets in the coastal atmosphere: Potential daytime marine sources. *Atmos. Chem. Phys.* **23**, 14761–14778 (2023).

43. A. Saiz-Lopez, R. von Glasow, Reactive halogen chemistry in the troposphere. *Chem. Soc. Rev.* **41**, 6448–6472 (2012).

44. X. Liu, H. Qu, L. G. Huey, Y. Wang, S. Sjostedt, L. Zeng, K. Lu, Y. Wu, M. Ho, M. Shao, T. Zhu, Y. Zhang, High levels of daytime molecular chlorine and nitryl chloride at a rural site on the North China plain. *Environ. Sci. Technol.* **51**, 9588–9595 (2017).

45. Y. Li, G. Shi, Z. Chen, Spatial and temporal distribution characteristics of ground-level nitrogen dioxide and ozone across China during 2015–2020. *Environ. Res. Lett.* **16**, 124031 (2021).

46. C. Xue, C. Zhang, C. Ye, P. Liu, V. Catoire, G. Krysztofiak, H. Chen, Y. Ren, X. Zhao, J. Wang, F. Zhang, C. Zhang, J. Zhang, J. An, T. Wang, J. Chen, J. Kleffmann, A. Mellouki, Y. Mu, HONO budget and its role in nitrate formation in the rural North China plain. *Environ. Sci. Technol.* **54**, 11048–11057 (2020).

47. B. H. Lee, F. D. Lopez-Hilfiker, C. Mohr, T. Kurten, D. R. Worsnop, J. A. Thornton, An iodide-adduct high-resolution time-of-flight chemical-ionization mass spectrometer: Application to atmospheric inorganic and organic compounds. *Environ. Sci. Technol.* **48**, 6309–6317 (2014).

48. A. K. Tobler, A. Skiba, D. S. Wang, P. Croteau, K. Styszko, J. Necki, U. Baltensperger, J. G. Slowik, A. S. H. Prevot, Improved chloride quantification in quadrupole aerosol chemical speciation monitors (Q-ACSMs). *Atmos. Meas. Tech.* **13**, 5293–5301 (2020).

49. Y. Chen, Y. Tan, P. Zheng, Z. Wang, Z. Zou, K.-F. Ho, S. Lee, T. Wang, Effect of NO_2 on nocturnal chemistry of isoprene: Gaseous oxygenated products and secondary organic aerosol formation. *Sci. Total Environ.* **842**, 156908 (2022).

50. H. Zhao, F. Yang, Z. Wang, Y. Li, J. Guo, S. Li, J. Shu, M. Chen, Chlorine and heavy metals removal from municipal solid waste incineration fly ash by electric field enhanced oxalic acid washing. *J. Environ. Manage.* **340**, 117939 (2023).

51. Z. Zong, C. Ren, X. Shi, Z. Sun, X. Huang, C. Tian, J. Li, G. Zhang, Y. Fang, H. Gao, Isotopic comparison of ammonium between two summertime field campaigns in 2013 and 2021 at a background site of North China. *Sci. Total Environ.* **905**, 167304 (2023).

52. H. Zhou, A. Meng, Y. Long, Q. Li, Y. Zhang, A review of dioxin-related substances during municipal solid waste incineration. *Waste Manag.* **36**, 106–118 (2015).

53. A. Cincinelli, F. Pieri, Y. Zhang, M. Seed, K. C. Jones, Compound specific isotope analysis (CSIA) for chlorine and bromine: A review of techniques and applications to elucidate environmental sources and processes. *Environ. Pollut.* **169**, 112–127 (2012).

54. M. Filippini, I. Nijenhuis, S. Kümmel, V. Chiarini, G. Crosta, H. Richnow, A. Gargini, Multi-element compound specific stable isotope analysis of chlorinated aliphatic contaminants derived from chlorinated pitches. *Sci. Total Environ.* **640**, 153–162 (2018).

55. M. Zhang, H. Wei, J. Lu, A. E. Williams-Jones, C. J. Eastoe, W. Li, Z. Xia, S. V. Hohl, J. Ma, Y. Cai, S. Jiang, J. Wang, Estimates of chlorine isotope fractionation factors using density functional theory: Applications to ore-forming systems. *Geochim. Cosmochim. Acta* **367**, 1–15 (2024).

56. M. Xia, T. Wang, Z. Wang, Y. Chen, X. Peng, Y. Huo, W. Wang, Q. Yuan, Y. Jiang, H. Guo, C. Lau, K. Leung, A. Yu, S. Lee, Pollution-derived Br_2 boosts oxidation power of the coastal atmosphere. *Environ. Sci. Technol.* **56**, 12055–12065 (2022).

57. M. G. Hastings, J. C. Jarvis, E. J. Steig, Anthropogenic impacts on nitrogen isotopes of ice-core nitrate. *Science* **324**, 1288–1288 (2009).

58. K. H. Hamed, A. R. Rao, A modified Mann-Kendall trend test for autocorrelated data. *J. Hydrol.* **204**, 182–196 (1998).

59. M.-T. Leu, R. S. Timonen, L. F. Keyser, Y. L. Yung, Heterogeneous reactions of HNO_3(g) + NaCl(s) → HCl(g) + $NaNO_3$(s) and N_2O_5(g) + NaCl(s) → $ClNO_2$(g) + $NaNO_3$(s). *J. Phys. Chem. Lett.* **99**, 13203–13212 (1995).

60. D. Curran-Everett, Explorations in statistics: Standard deviations and standard errors. *Adv. Physiol. Educ.* **32**, 203–208 (2008).

61. A. Ding, T. Wang, C. Fu, Transport characteristics and origins of carbon monoxide and ozone in Hong Kong, South China. *J. Geophys. Res. Atmos.* **118**, 9475–9488 (2013).

62. Z. Zong, T. Wang, J. Chai, Y. Tan, P. Liu, C. Tian, J. Li, Y. Fang, G. Zhang, Quantifying the nitrogen sources and secondary formation of ambient HONO with a stable isotopic method. *Environ. Sci. Technol.* **57**, 16456–16464 (2023).

63. J. Stutz, R. Ackermann, J. D. Fast, L. Barrie, Atmospheric reactive chlorine and bromine at the Great Salt Lake, Utah. *Geophys. Res. Lett.* **29**, 18-1–18-4 (2002).

64. C. Hoermann, H. Sihler, S. Beirle, M. P. de Vries, U. Platt, T. Wagner, Seasonal variation of tropospheric bromine monoxide over the Rann of Kutch salt marsh seen from space. *Atmos. Chem. Phys.* **16**, 13015–13034 (2016).

65. H. Zhong, R.-J. Huang, Y. Chang, J. Duan, C. Lin, Y. Chen, Enhanced formation of secondary organic aerosol from photochemical oxidation during the COVID-19 lockdown in a background site in Northwest China. *Sci. Total Environ.* **778**, 144947 (2021).

66. Y. Ji, G. Huey, D. J. Tanner, Y. R. Lee, P. R. Veres, J. A. Neuman, Y. Wang, X. Wang, A vacuum ultraviolet ion source (VUV-IS) for iodide-chemical ionization mass spectrometry: A substitute for radioactive ion sources. *Atmos. Meas. Tech.* **13**, 3683–3696 (2020).

67. K. Song, S. Guo, H. Wang, Y. Yu, H. Wang, R. Tang, S. Xia, Y. Gong, Z. Wan, D. Lv, R. Tan, W. Zhu, R. Shen, X. Li, X. Yu, S. Chen, L. Zeng, X. Huang, Measurement report: Online measurement of gas-phase nitrated phenols utilizing a CI-LToF-MS: Primary sources and secondary formation. *Atmos. Chem. Phys.* **21**, 7917–7932 (2021).

68. J. Xue, Z. Yuan, A. K. H. Lau, J. Z. Yu, Insights into factors affecting nitrate in $PM_{2.5}$ in a polluted high NO_x environment through hourly observations and size distribution measurements. *J. Geophys. Res. Atmos.* **119**, 4888–4902 (2014).

69. C. Lin, R.-J. Huang, J. Duan, H. Zhong, W. Xu, Primary and secondary organic nitrate in Northwest China: A case study. *Environ. Sci. Technol. Lett.* **8**, 947–953 (2021).

70. A. Mattei, F. Huneau, E. Garel, S. Santoni, T. Leydier, Y. Vystavna, Gross primary production of Mediterranean watersheds: Using isotope mass balance approach to improve estimations. *Ecohydrology* **17**, e2619 (2024).

71. T. E. Graedel, W. C. Keene, The budget and cycle of Earth's natural chlorine. *Pure Appl. Chem.* **68**, 1689–1697 (1996).

72. T. Svensson, H. Kylin, M. Montelius, P. Sanden, D. Bastviken, Chlorine cycling and the fate of Cl in terrestrial environments. *Environ. Sci. Pollut. Res.* **28**, 7691–7709 (2021).

73. S. Atashgahi, M. G. Liebensteiner, D. B. Janssen, H. Smidt, A. J. M. Stams, D. Sipkema, Microbial synthesis and transformation of inorganic and organic chlorine compounds. *Front. Microbiol.* **9**, 3079 (2018).

74. Y. N. Vodyanitskii, M. I. Makarov, Organochlorine compounds and the biogeochemical cycle of chlorine in soils: A review. *Eurasian Soil Sci.* **50**, 1025–1032 (2017).

75. J. G. Schilling, C. K. Unni, M. L. Bender, Origin of chlorine and bromine in the oceans. *Nature* **273**, 631–636 (1978).

76. M. A. Kendrick, C. Hemond, V. S. Kamenetsky, L. Danyushevsky, C. W. Devey, T. Rodemann, M. G. Jackson, M. R. Perfit, Seawater cycled throughout Earth's mantle in partially serpentinized lithosphere. *Nat. Geosci.* **10**, 222–228 (2017).

77. S. L. Gong, A parameterization of sea-salt aerosol source function for sub- and super-micron particles. *Global Biogeochem. Cycles* **17**, 1097 (2003).

78. R. A. Zaveri, R. C. Easter, J. D. Fast, L. K. Peters, Model for simulating aerosol interactions and chemistry (MOSAIC). *J. Geophys. Res.* **113**, D13204 (2008).

79. M. L. Wesely, Parameterization of surface resistances to gaseous dry deposition in regional-scale numerical models. *Atmos. Environ.* **41**, 52–63 (2007).

80. G. A. Grell, D. Dévényi, A generalized approach to parameterizing convection combining ensemble and data assimilation techniques. *Geophys. Res. Lett.* **29**, 38-1–38-4 (2002).

81. A. Badia, C. E. Reeves, A. R. Baker, A. Saiz-Lopez, R. Volkamer, T. K. Koenig, E. C. Apel, R. S. Hornbrook, L. J. Carpenter, S. J. Andrews, T. Sherwen, R. von Glasow, Importance of reactive halogens in the tropical marine atmosphere: A regional modelling study using WRF-Chem. *Atmos. Chem. Phys.* **19**, 3161–3189 (2019).

82. M. J. Iacono, J. S. Delamere, E. J. Mlawer, M. W. Shephard, S. A. Clough, W. D. Collins, Radiative forcing by long-lived greenhouse gases: Calculations with the AER radiative transfer models. *J. Geophys. Res.* **113**, D13103 (2008).

83. F. Chen, J. Dudhia, Coupling an advanced land surface-hydrology model with the Penn State-NCAR MM5 modeling system. Part II: Preliminary model validation. *Mon. Weather Rev.* **129**, 587–604 (2001).

84. H. Morrison, G. Thompson, V. Tatarskii, Impact of cloud microphysics on the development of trailing stratiform precipitation in a simulated squall line: Comparison of one- and two-moment schemes. *Mon. Weather Rev.* **137**, 991–1007 (2009).

85. S. Sukoriansky, B. Galperin, V. Perov, Application of a new spectral theory of stably stratified turbulence to the atmospheric boundary layer over sea ice. *Boundary Lay. Meteor.* **117**, 231–257 (2005).

86. L. K. Emmons, S. Walters, P. G. Hess, J. F. Lamarque, G. G. Pfister, D. Fillmore, C. Granier, A. Guenther, D. Kinnison, T. Laepple, J. Orlando, X. Tie, G. Tyndall, C. Wiedinmyer, S. L. Baughcum, S. Kloster, Description and evaluation of the model for ozone and related chemical tracers, version 4 (MOZART-4). *Geosci. Model Dev.* **3**, 43–67 (2010).

87. X. X. Tie, S. Madronich, S. Walters, R. Y. Zhang, P. Rasch, W. Collins, Effect of clouds on photolysis and oxidants in the troposphere. *J. Geophys. Res.* **108**, 4642 (2003).
